# A comprehensive guide to the Argentinian case-bearer beetle fauna (Coleoptera, Chrysomelidae, Camptosomata)

**DOI:** 10.3897/zookeys.677.10778

**Published:** 2017-05-26

**Authors:** Federico A. Agrain, Maria Lourdes Chamorro, Nora Cabrera, Davide Sassi, Sergio Roig-Juñent

**Affiliations:** 1 Laboratorio de Entomología, IADIZA (CCT-Mendoza), Consejo Nacional de Investigaciones Científicas y Técnicas (CONICET), C.C. 507, 5500 Mendoza, Argentina; 2 Department of Entomology, National Museum of Natural History, Smithsonian Institution, Systematic Entomology Laboratory, USDA, NMNH, Smithsonian Institution, P.O. Box 37012, MRC-187, Washington, DC, 20013-7012, USA; 3 División Entomología. Museo de La Plata. Paseo del Bosque s/n, 1900 La Plata, Argentina; 4 Centro di Entomologia Alpina – Università degli Studi di Milano, Via Celoria 2, 20133 Milano, Italy

**Keywords:** Argentina, Cryptocephalinae, Distribution, Diversity, Genera, Lamprosomatinae, Provinces, Richness, Taxonomy

## Abstract

Knowledge of Argentinian Camptosomata has largely remained static for the last 60 years since the last publication by Francisco de Asis Monrós in the 1950’s. One hundred and ninety Camptosomata species (182 Cryptocephalinae and 8 Lamprosomatinae) in 31 genera are recorded herein from Argentina. Illustrated diagnostic keys to the subfamilies, tribes, subtribes and genera of Argentinian Camptosomata, plus species checklists and illustrations for all genera of camptosomatan beetles cited for each political region of Argentina are provided. General notes on the taxonomy and distribution, as well as basic statistics, are also included. This study provides basic information about the Camptosomata fauna in Argentina that will facilitate in the accurate generic-level identification of this group and aid subsequent taxonomic revisions, and phylogenetic, ecological, and biogeographic studies. This information will also facilitate faunistic comparisons between neighboring countries. Two nomenclatural acts are proposed: Temnodachrys (Temnodachrys) argentina (Guérin, 1952), **comb. n.**, and *Metallactus bivitticollis* (Jacoby, 1907), **comb. n.** The following are new records for Argentina: *Stegnocephala xanthopyga* (Suffrian, 1863) and *Lamprosoma azureum* Germar, 1824. Currently, the most diverse camptosomate tribe in Argentina is Clytrini, with almost twice the number of species of Cryptocephalini. New records for Argentina are predicted.

## Introduction

In Argentina there are 956 recorded species of Chrysomelidae (excluding Bruchinae) in 258 genera (Cabrera and Roig-Juñent 1998a). Only two subfamilies of leaf beetles are not represented in Argentina: Donaciinae Kirby and Synetinae LeConte and Horn. Most subtropical species are distributed in the Amazonian and Chacoan domain (Cabrera and Willink 1973). However, current knowledge of Argentinian Chrysomelidae is incomplete. The present paper is the first one in a planned series on the Argentine chrysomelid fauna.

The chrysomelid subfamilies Cryptocephalinae and Lamprosomatinae are collectively known as “Camptosomata” or “case-bearers,” due to the peculiar habit of having their eggs, larvae, and pupae living in a fecal protective case (Brown and Funk 2005; Chaboo et al. 2008; Erber 1988, Jolivet and Hawkeswood 1995). Adults of case-bearing chrysomelids feed on foliage of a variety of eudicots (Erber 1988), but their larvae often show departures from strict phytophagy. The larvae of some Clytrini and Cryptocephalini live in ant nests, where they feed on other items such as ant droppings and pellets, detritus, leaf litter and even dead insects collected by the ants (Agrain et al. 2015, and references therein). The larvae of camptosomates can be easily recognized by the behavior of carrying a portable case and the J-shaped body morphology.

Lamprosomatinae includes four tribes (Chamorro and Konstantinov 2011): Cachiporrini (1 genus), Neochlamysini (2 genera), Sphaerocharini (1 genus), and Lamprosomatini (10 genera) (Seeno and Wilcox 1982), totalling 190 described species (Reid 2016). Reid (2016) and Chamorro (2014a), concur on a world estimate of 250 species. In Argentina, the only genus represented is *Lamprosoma* Kirby. Cryptocephalinae includes ~5300 species, independently calculated by Chamorro, (2014b) and Reid (2016) that are classified into three tribes: Cryptocephalini, Clytrini, and Fulcidacini (until recently treated under the name Chlamisini) as originally proposed by Reid (1995, 2000). Members of the subfamily are distributed worldwide, but many tribes have distinct distributions (Erber 1988). Species are phytophagous in the adult stage, primarily leaf and flower feeders. All three tribes of this subfamily have representative genera in Argentina. The main goal of this contribution is to provide an updated systematic framework for Argentinian Camptosomata, treating all of its genera in order to better measure our current knowledge of these groups. This work includes the compilation of former fragmentary literature on the subject.

### Type material of Argentinian Camptosomata

Most of the type specimens of Argentinian Camptosomata are deposited in European institutions: The Natural History Museum, London, United Kingdom (BMNH), Hungarian Natural History Museum, Budapest, Hungary (HNHM), Institut Royal des Sciences Naturelles de Belgique, Brussels, Belgium (KBIN), Museo Regionale di Scienze Naturali di Torino (MRSN), Museum für Naturkunde der Humbolt Universitat, Berlin, Germany (ZMHB), and National Museum, Prague, Czech Republic (NMPC). There are also type specimens in institutions in the United States: Museum of Comparative Zoology Collection, Harvard University, Boston, USA (MCZ), and the National Museum of Natural History, Smithsonian Institution, Washington D.C., USA (USNM). Yet, some type specimens are deposited in Museo de La Plata, La Plata, Argentina (MLPA) (see Cabrera and Roig-Juñent 1998b), Instituto y Fundación Miguel Lillo, Tucumán, Argentina (IMLA) (Aranda et al. 2016), and Museo Argentino de Ciencias Naturales ‘Bernardino Rivadavia’, Buenos Aires, Argentina (MACN) (Bachmann and Cabrera 2010). Two of the most prominent workers on Argentinian Camptosomata were Francisco de Asis Monrós, whose collection was donated to the Smithsonian Institution (USNM) (Staines 1995), and Manuel Viana, whose collection is now housed in Tucumán and Salta Provinces in Argentina and in Chile. More recently, a few type specimens have been deposited in in the Instituto Argentino de Investigaciones de las Zonas Áridas, Mendoza, Argentina (IADIZA) by Agrain (2013, 2014).

## Methods

We studied all catalogs and specialized literature dealing with the genera treated in this contribution. Nomenclature follows previous authors, especially those who made extensive revisions of this group, such as Andrew Moldenke, Francisco Monrós, Jacintho Guérin, and Martin Jacoby. Characters used for identification keys are those used by: Agrain and Roig-Juñent (2011), Chamorro-Lacayo and Konstantinov (2009), Guérin (1943), Karren (1972), Lacordaire and Chapuis (1854), Moldenke (1970, 1981), Monrós (1949a, 1953a), and Riley et al. (2002). An identification key to the subfamilies, tribes, subtribes, genera, and subgenera of Argentinian Camptosomata was made by compiling and modifying previous publications as indicated in Table 1. Some couplets in our key, derived from keys provided by earlier authors, are based on extreme representatives of a rather continuous spectrum. The latter is due to the fact that many genera, and especially subgenera, require modern revision. Our key is built for the identification of taxa on the territory of Argentina but is useful for the South American continent. The characters given for some widely distributed genera (e.g., *Cryptocephalus* Geoffroy, *Pachybrachis* Chevrolat) may not apply to species outside Argentina. Images of dorsal and lateral habiti were taken by different authors as indicated in superscript values: (^1^) F. Agrain, (^2^) L. Chamorro, (^3^) C. Gorretta, N. Cabrera, and (^4^) D. Sassi, and edited by F. Agrain.

**Table 1.** Main sources of information used for the identification key.

Group	Citation
Genera of Fulcidacini	Chamorro-Lacayo and Konstantinov (2009), Karren (1972), Lacordaire and Chapuis (1854).
Genera and subgenera of Clytrini	Agrain and Roig-Juñent (2011), Lacordaire and Chapuis (1854), Moldenke (1981), Monrós (1953a).
Subtribes of Cryptocephalini	Lacordaire and Chapuis (1854), Riley et al. (2002).
Genera of Cryptocephalina	Lacordaire and Chapuis (1854), Monrós (1949a), Watts (2005).
Genera of Pachybrachina	Chamorro (2013); Lacordaire and Chapuis (1854).

**Figure 1.**
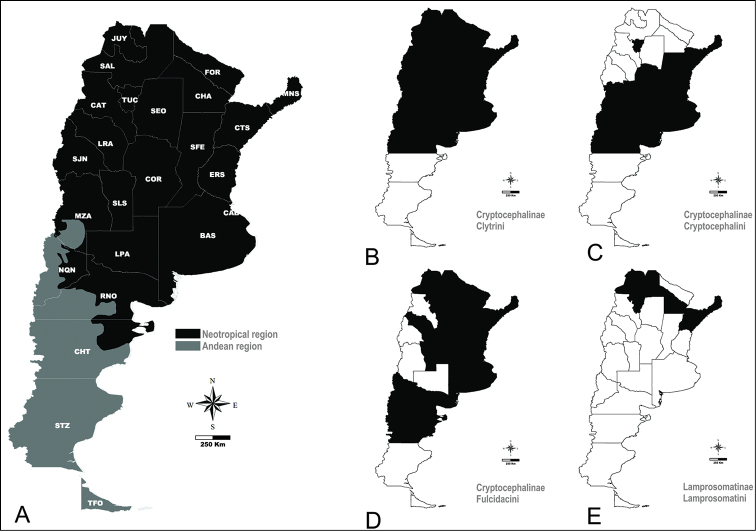
Distribution of Camptosomata tribes. **A** Map showing Argentinian administrative divisions with abbreviation as used in text. Andean and Neotropical regions as indicated in color reference **B** In black, administrative divisions containing Clytrini species **C** In black, administrative provinces containing Cryptocephalini species **D** In black, administrative divisions containing Fulcidacini species **E** In black, administrative divisions containing Lamprosomatini species.

We conducted an exhaustive search of all publications citing Argentinian camptosomates. Here we present a checklist of all currently known camptosomate species from Argentina, their distribution, host plant preferences, juvenile data where available, and known predators. Junior synonyms are provided for each species when applicable. The 24 provinces in Argentina (Fig. 1A) are abbreviated as follows: Buenos Aires (BAS), Catamarca (CAT), Chaco (CHA), Chubut (CHT), Ciudad Autónoma de Buenos Aires (CAB), Córdoba (COR), Corrientes (CTS), Entre Ríos (ERS), Formosa (FOR), Jujuy (JUY), La Pampa (LPA), La Rioja (LRA), Mendoza (MZA), Misiones (MNS), Neuquén (NQN), Río Negro (RNO), Salta (SAL), San Juan (SJN), San Luis (SLS), Santa Cruz (SCZ), Santa Fe (SFE), Santiago del Estero (SEO), Tierra del Fuego (TFO), Tucumán (TUC). The source map of Andean and Neotropical regions of Argentina was obtained from Löwenberg-Neto (2014).

### Terminology

Terminology follows previous authors as indicated in table 1. The term “egg anal pit” refers to a medioventral excavation on terminal abdominal ventrite, mostly present in the females (Chaboo 2007, and references therein). The term “rectal apparatus” refers to a specialized region of the rectum of females, whose sclerites and muscles are used during oviposition (De Monte 1957, Erber 1988, Brown and Funk 2005, Schöller 2008). This structure is unique to the Cryptocephalinae (Reid 1995).

## Results

### Keys to the subfamilies, tribes, subtribes, genera, and subgenera of Argentinian Camptosomata

Note: Some of the characters in this key are valid for Neotropical species only

**Table:** 

1	Body dorsally highly convex and ventrally flattened (semicircular in cross-section) (Fig. 2A); abdominal ventrites not connate; antennal grooves present on intercoxal prosternal process; females without a well developed fovea (egg anal pit) on ventrite V (**Lamprosomatinae**). Distal margin of last ventrite thick (Fig. 2B); last ventrite not excised in shape of arc (Fig. 2C); pygidium completely covered by elytra; scutellum acutely triangular (small to very small); elytral punctation arranged in regular rows or with a tendency to form such rows	***Lamprosoma* Kirby** (Fig. 34). **(Lamprosomatini)**
–	Body not highly convex, not flattened ventrally (Fig. 2D) (oval in cross-section); abdominal ventrites connate; antennal grooves absent on intercoxal prosternal process (except Fulcidacini and Ischiopachina (Clytrini); females with a distinct, variably shaped fovea (egg anal pit) on ventrite V	**2. (Cryptocephalinae)**
**Cryptocephalinae**
2(1)	Intercoxal prosternal process with antennal furrows; body surface usually tuberculate (Fig. 2E); elytral suture usually serrate	**4 (Fulcidacini)**
2’	Pronotum and intercoxal prosternal process without antennal furrows (Ischiopachina with furrows on hypomeron); body surface not tuberculate; elytral suture entire	**3**
3(2)	Antennae short (not surpassing the length of pronotum), serrate; procoxae contiguous (Fig. 2F)	**9 (Clytrini)**
3’	Antennae long (surpassing the length of pronotum, sometimes nearly equal to total body length), filiform, sometimes some segments expanded and flattened; procoxae separated (Fig. 2G)	**29 (Cryptocephalini)**
**Fulcidacini**
4(2)	Intercoxal prosternal process nearly rectangular, with posterior margin slightly narrower than anterior margin; pronotal and elytral tubercles reduced, sometimes with velvet patches	***Melittochlamys* Monrós** (Fig. 32)
4’	Intercoxal prosternal process varying in shape, triangular or angulate between mesocoxae, but never rectangular, with posterior margin much narrower than anterior margin; pronotal and elytral tubercles well developed	**5**
5(4)	Body equal to or greater than 10 mm long; colour brightly metallic; head with vertex longitudinally impressed; tarsal claws simple	***Fulcidax* Voet** (Fig. 31)
5’	Body less than 10 mm long; head with vertex not impressed; tarsal claws usually appendiculate (except *Exema* (from simple to appendiculate)	**6**
6(5)	Pronotum with six distinct, small, sharp, longitudinal carinae converging posteromedially, fan-like; color uniform, generally black	***Aulacochlamys* Monrós** (Fig. 28)
6’	Pronotum with or without tubercles, but never with six longitudinal, fan-like carinae	**7**
7(6)	Head not completely retracted into the prothorax; mandibles in males larger than in females; intercoxal prosternal process strongly and abruptly constricted behind anterior margin; prosternal process more than 3/4 as long as intercoxal prosternal process (Fig. 2H) …***Pseudochlamys* Lacordaire** (Fig. 33)
7’	Head completely retracted into prothorax; mandibles in males as large as in females; intercoxal prosternal process gradually constricted at about 2/3 of its length (Fig. 2I)	**8**
8(7)	Males without spines or spinulae on ventrite I; antennomere V nearly as long as VI; elytral suture crenulation usually incomplete (*i.e.*, suture entire immediately following scutellum)	***Chlamisus* Rafinesque** (Fig. 29)
8’	Males with spines or spinulae on ventrite I; antennomere V much shorter than VI; elytral suture crenulation always complete	***Exema* Lacordaire** (Fig. 30)
**Clytrini**
9(3)	Prothorax with lateral antennal grooves on hypomeron **Ischiopachina Chapuis** (Monotypic subtribe). Elytra without strong parallel longitudinal carinae; color metallic	***Ischiopachys* Chevrolat** (Fig. 16)
9’	Prothorax without lateral antennal grooves on edge of hypomeron	**10**
10(9)	Tarsal claws simple	**12 (Megalostomina Chapuis)**
10’	Tarsal claws bifid or appendiculate (Fig. 3A)	**11**
11(10)	Scutellum flat, in the same horizontal plane as elytra; elytra without longitudinal carinae, frons distinct	**15 (Babiina Chapuis)**
11’	Scutellum raised above elytral plane; elytra with strong parallel, longitudinal carinae; frons very narrow **Arateina Moldenke**; (monogeneric subtribe)	***Aratea* Lacordaire** (Fig. 4). (monogeneric subtribe)
12(10)	Eyes entire or only slightly emarginate	**13**
12’	Eyes strongly emarginate	***Megalostomis* Chevrolat** (Fig. 19)
13(12)	Scutellum foveate; body not metallic; pygidium with transverse subapical carina; aedeagus occupying entire abdominal length	***Euryscopa*** (***Coleomonrosa***) **Moldenke** (Fig. 18)
13’.	Scutellum not foveate; body brilliant metallic, or with noticeable metallic reflections; pygidium without subapical transverse carina; aedeagus smaller, not occupying entire abdominal length	**14**
14(13)	Eyes elongate, not protruding; body robust, subquadrate	***Themesia* Lacordaire** (Fig. 20)
14’	Eyes round and protruding; body elongate	***Coscinoptera* Lacordaire** (Fig. 17)
15(11)	Tarsal claws bifid; dorsum of body uniformly metallic; body lengthened	***Helioscopa* Gistel** (Fig. 9)
15’	Tarsal claws appendiculate; dorsum of body not uniformly metallic; body compact	**16**
16(15)	Epipleural lobes very pronounced;angle of epipleural lobes rounded; elytra striae strongly impressed; dorsal coloration black, with omnipresent metallic bronze reflections	**Saxinis (Saxinis) Lacordaire** (Fig. 13)
16’	Epipleural lobes weakly developed, not angulate, no more than two striae wide; elytra striae not strongly impressed; dorsal coloration without bronze reflections	**17**
17(16)	Pygidium with transverse subapical angle, evenly bent perpendicular to longitudinal axis of body; epipleural lobe not well developed in lateral view	**18**
17’	Pygidium flat, surface slightly convex, with tip sometimes bent; epipleural lobe distinctly rounded in lateral view	**23**
18(17)	Eyes feebly emarginate, distinctly projecting, conspicuously protruding at sides of head	***Dinophthalma* Lacordaire** (Fig. 8)
18’	Eyes distinctly emarginate, not markedly projecting	**19**
19(18)	Anterior margin of pronotum arcuate, entirely covering head from dorsal view; body shape elongate, cylindrical and flat; size large, greater than 10 mm long; frons flat; lateral margins of prothorax not widely explanate	**Babia (Coleolacordairei) Moldenke** (Fig. 5)
19’	Anterior margin of pronotum transverse or arcuate, but not concealing entire head from dorsal view	**20**
20(19)	Body shape strongly cylindrical, elongate, not flattened; elytra not fully covering pygidium; elytral punctation barely noticeable	***Cylindrodachrys* Monrós** (Fig. 6)
20’	Body shape not cylindrical or elongate; elytra fully covering pygidium; elytral punctation evident	**21**
21(20)	Frons strongly tapering, triangular, without transverse sulcus; body shape subquadrate; aedeagus with strong dorsal and ventral tufts of pubescence	***Pnesthes* Lacordaire** (Fig. 11)
21’	Frons not strongly tapering below eyes, subrectangular, with length only slightly greater than width; aedeagus without pronounced ventral and dorsal patches of setae, with only a few dorsal setae present	**22**. ***Temnodachrys* Monrós** (Fig. 14)
22(21)	Frons with deep transverse sulcus (Fig. 3B); body shape subrectangular	**Temnodachrys (Temnodachrys) Monrós**
22’	Frons without deep transverse sulcus; body guttiform or minute and with subparallel sides	**Temnodachrys (Eudachrys) Monrós**
23(17)	Body shape subcircular in outline; legs with longitudinal carinae; anterior pronotal margin strongly explanate and completely concealing head from dorsal view	**24**
23’	Body shape subrectangular, sides subparallel; legs without longitudinal carinae; anterior margin of pronotum never concealing all of head in dorsal view	**27**
24(23)	Forelegs longer (especially in males) than mid- and hind legs; tarsomere III enlarged, shallowly excavated; head less reflexed, 90º with respect to prosternum (Fig. 3C)	***Stereoma* Lacordaire** (Fig. 12)
24’	All legs with similar development; tarsomere III narrow, deeply excavated; head more reflexed, forming 45º angle with respect to prosternum (Fig. 3D)	**25**. ***Urodera* Lacordaire** (Fig. 15)
25(24)	Posterior margin of pronotum broadly expanded, forming distinct scutellar lobe with angular corners (Fig. 3E)	**Urodera (Austrurodera) Moldenke**
25’	Posterior margin of pronotum not broadly expanded and not forming a scutellar lobe with angular corners	**26**
26(25)	Front tibiae with indistinct posterolateral carinae, with surface not deeply excavate and reflective between carinae; frons of male with three shallow depressions	**Urodera (Urodera) Lacordaire**
26’	Front tibiae with strong posterolateral carinae, with surface deeply excavate and reflective between carinae; frons of male with deep medial depression	**Urodera (Stereomoides) Moldenke**
27(23)	Pronotum with weak metallic green reflections; antennomere IV much smaller than V; frons wide, with width greater than or subequal to length; frons without medial pit	**28**. ***Paraurodera* Moldenke**. (Fig. 10).
27’	Pronotum without metallic reflections; antennomere IV subequal in size to V; frons narrow, with length more than twice width; frons with deep medial pit	***Dachrys* Erichson** (Fig. 7)
28(27)	Anterior margin of pronotum transverse, not concealing head at all in dorsal view; frons with submedial depressions; sexual dimorphism of frons extreme, the male having extremely wide frons and elongate mandibles	**Paraurodera (Torourodera) Moldenke**
28’	Anterior margin of pronotum explanate and partially concealing head; frons with medial and two submedial depressions; sexual dimorphism reduced, with frons and mandibles similarly developed in male and female	**Paraurodera (Paraurodera) Moldenke**
**Cryptocephalini**
29(3)	Claws simple or, if appendiculate, intercoxal prosternal process longer than wide to subquadrate	**30**
29’	Claws appendiculate, each with broad, basal tooth; intercoxal prosternal process wider than long	**31. Monachulina Leng**
30(29)	Pronotum margined at base (except in *Mylassa*), not crenulate (Fig. 3F)	**32** (**Pachybrachina Chapuis)**
30’	Pronotum not margined at base, usually crenulate (Fig. 3G) (**Cryptocephalina Gyllenhal)**. Eyes with distinct excavation on internal margin; dorsum glabrous; male front tibiae with reduced sexual dimorphism; posterior pronotal margin not produced	***Cryptocephalus* Geoffroy** (Fig. 21)
31(29)	Anterior margin of pronotum simple, arcuate; pronotal punctures distinct throughout; intercoxal prosternal process bilobed, with small lateral projections; anterior margin of prosternum uniformly concave; pronotal anterior opening circular	***Lexiphanes* Gistel** (Fig. 22)
31’	Anterior margin of pronotum produced; pronotal punctures absent; intercoxal prosternal process truncate; anterior margin of prosternum with one or two medial flanges; pronotal anterior opening ventrally widened	***Stegnocephala* Baly** (Fig. 23)
32(30)	Eyes small, bulging, with canthus shallow	**33**
32’	Eyes large, extending dorsad beyond upper third of head, usually with upper half of eye larger than ventral half; canthus deep, extending approximately 1/4 distance into eye; posterior margin of pronotum (directly opposite scutellum) not produced posteriorly, margined with basal row of punctures, bi-sinuate; scutellum not heart-shaped	**34**
33(32)	Dorsal surface generally setose; pronotum greatly vaulted, with lateral margins narrow; pronotum medially lobed posteriorly, lobe elevated and truncate; scutellum heart-shaped	***Mylassa* Stål** (Fig. 26)
33’	Dorsal surface glabrous; pronotum regularly convex, with lateral margins prominent, visible from above, with posterior margin regularly biconcave, with mesobasal region regularly rounded and slightly produced posterad; scutellum with posterior margin truncate	***Ambrotodes* Suffrian** (Although this genus has not been yet reported from Argentina, we include it in this key because its species are common along the eastern border of the Andes in Chile.
34(32)	Posterior margin of intercoxal prosternal process convex, produced beyond posterior margin of prothorax; mesotibial spurs present or absent; body robust; punctures not deep or large, particularly on pronotum; dorsal surface shiny	**35**
34’	Posterior margin of intercoxal prosternal process straight, rarely produced beyond posterior margin of prothorax; gestalt cylindrical (height of each elytron approximately 2.5 width), pronotum narrower than elytral bases combined, overall flattened not vaulted; punctures on head, prothorax and elytra evident, large; elytral punctation commonly confused (but punctation in rows not uncommon); forefemora enlarged or not; each mesotibia usually with terminal spur in both sexes	***Pachybrachis* Chevrolat** (Fig. 27)
35(34)	Posterior margin of intercoxal prosternal process rounded; lateral margin of elytra deeply excised, exposing abdomen caudally; elytra length approximately 2× or less length of pronotum	***Griburius* Haldeman** (Fig. 24)
35’	Posterior margin of intercoxal prosternal process gradually narrowing, pointed; lateral edge of elytra not deeply excised; abdomen not exposed; elytral length greater than 2× length of pronotum	***Metallactus* Suffrian** (Fig. 25)

**Figure 2.**
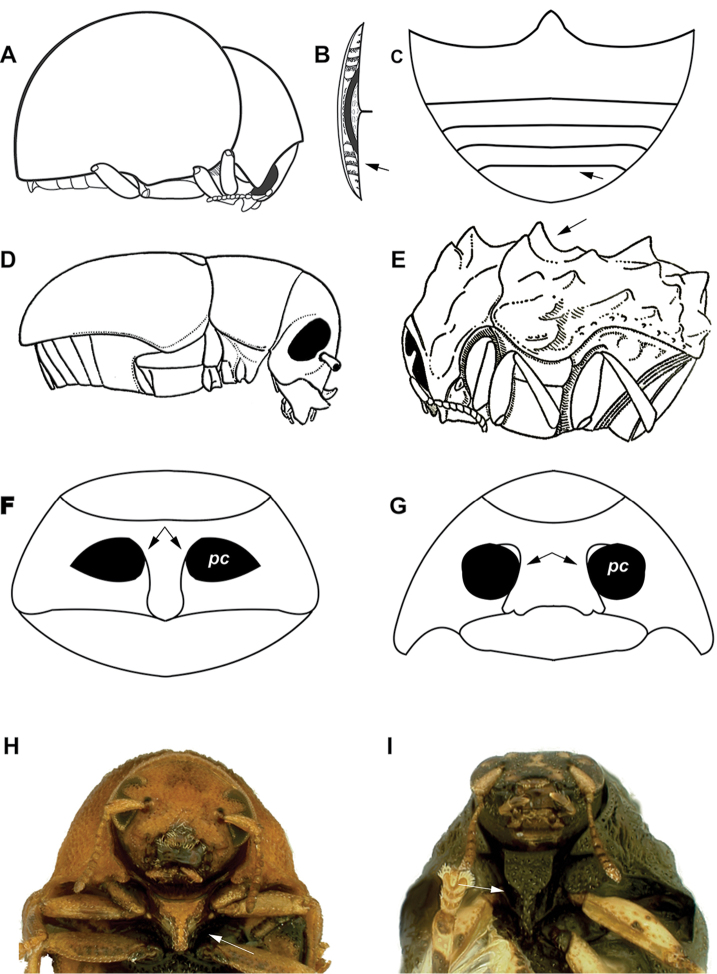
Diagnostic characters plate 1. **A** Body dorsally highly convex and ventrally flattened (semicircular in cross-section (drawn after Monrós 1956) **B** Distal margin of last ventrite thick (drawn after Chamorro and Konstantinov 2011) **C** Last ventrite not excised in shape of arc **D** Body cylindrical, not flattened ventrally (drawn after Monrós 1953a) **E** body surface usually tuberculate (drawn after Monrós 1951) **F** procoxae prominent and contiguous **G** procoxae not prominent and separated **H** prosternal process more than 3/4 as long as intercoxal prosternal process (after Chamorro-Lacayo and Konstantinov (2009) **I** intercoxal prosternal process gradually constricted at about 2/3 of its length (after Chamorro-Lacayo and Konstantinov (2009).

**Figure 3.**
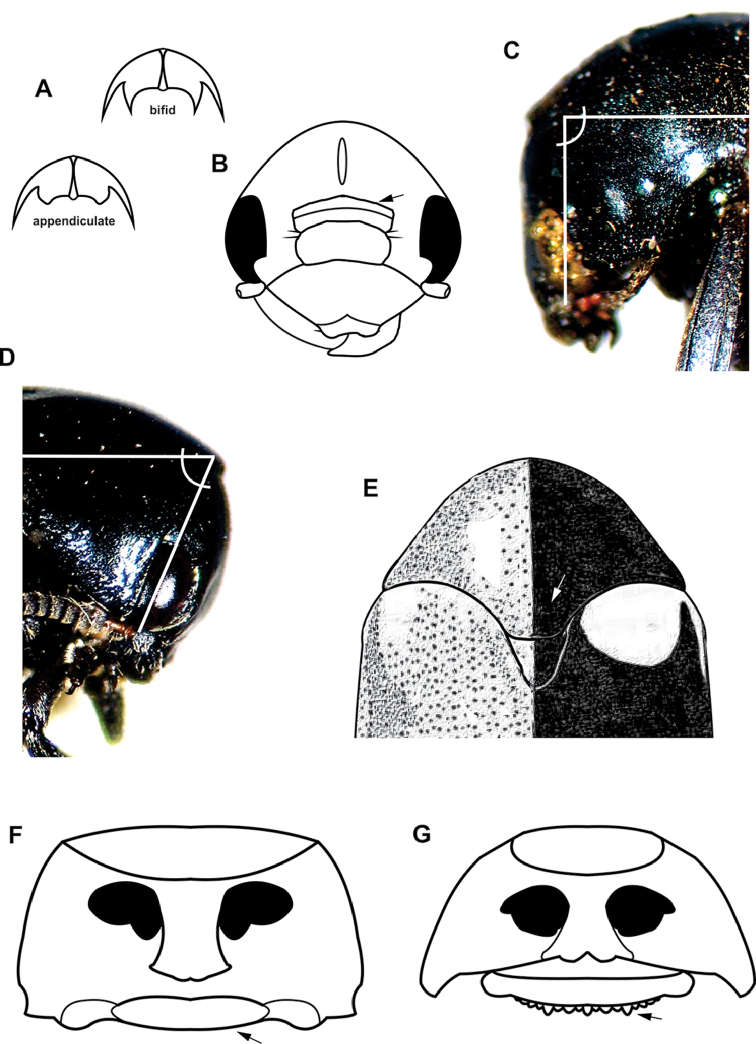
Diagnostic characters plate 2. **A** Tarsal claws bifid or appendiculate **B** Frons with deep transverse sulcus. **C** Head forming straight angle with respect to pronotum **D** Head strongly directed downward, forming 45º angle with respect to pronotum **E** Posterior margin of pronotum broadly expanded, forming distinct scutellar lobe with angular corners (drawn after Monrós 1953a) **F** Pronotum margined at base, not crenulate **G** Pronotum not margined at base, crenulate.

### Cryptocephalinae Gyllenhal, 1813

*Adults*: Body cylindrical, or rarely as long as wide; in dorsal view parallel-sided with prothorax mostly as wide as combined elytral bases; rarely body rounded; multicolored and patterned, particularly Cryptocephalini, black with red humeri commonly in Clytrini, brown, black, straw-yellow and some with velvet spots in Fulcidacini, glabrous to pubescent, particularly Clytrini. Head retracted into prothorax up to frons or almost completely, with compound eyes completely to barely visible from above. Compound eyes entire, level to strongly protuberant; canthus weak to deep. Antennae 11-segmented, longer than pronotum and filiform in Cryptocephalini (sometimes antennomeres distally dilated and flattened), shorter than pronotum and dentate in Clytrini, clavate in Fulcidacini. Pronotum about 0.75–1.0 times as long as wide, widest basally; sides slightly rounded or sinuate; base slightly narrower or as wide as combined elytral bases. Prosternum in front of coxae usually narrow and shorter than shortest diameter of a single coxal cavity, flat to moderately convex, sometimes produced to conceal mouthparts. Prosternal process complete, usually parallel-sided; notosternal sutures distinct. Procoxae not projecting below prosternum, without concealed lateral extensions; trochantins exposed within coxal cavity. Stridulatory device present on concealed part of mesoscutellum. Tarsi 5-5-5 in both sexes; penultimate tarsomere reduced and antepenultimate bilobed, all usually wider in males; tarsomere III densely clothed beneath with adhesive microtrichia; pretarsal claws simple to deeply bifid. Abdomen with five free ventrites and six tergites. Ventrite I more than twice as long as II, usually longer than ventrites II-IV combined, without postcoxal lines; intercoxal process narrowly rounded to almost truncate. Functional spiracles present on tergites I-VI. Tergite VI forming strongly pigmented pygidium, always exposed; anterior edge of sternite VIII in male without median strut. Ventrite V (=sternite VII) in females with variably-sized apical fovea. Males with segment IX membranous and spiculum gastrale Y-shaped. Aedeagus of cucujiform type; tegmen Y-shaped; struts (remnants of tergite IX) either present or absent; penis flattened to rounded, slightly to strongly curved apically; apically and/or laterally usually with tufts of setae. Sternite VIII in female lacking spiculum ventrale. Ovipositor short, rigid and oval with distinct proctigeral, paraproctal, and coxital baculi; paraprocts deltoid, slightly shorter than undivided coxites, sclerotized or less pigmented proximally, flattened, digitate lobes of variable form, apically setose; styli absent. Spermatheca strongly to moderately sclerotized, variably shaped, usually J-, C-, or S-shaped. Rectal sclerites (rectal apparatus) present in female.

*Larvae*: J-shaped, generally protected by a case. According to Reid (1990, 1995), Agrain and Marvaldi (2009) and Chamorro (2014b), the following features characterize the subfamily Cryptocephalinae in the broad sense (*i.e.*, including Clytrini, Cryptocephalini and Fulcidacini), and are probably synapomorphies of cryptocephalines, by outgroup comparison with other chrysomeloids and weevils (Reid 1995, 2000): body J-shaped; frons, clypeus, and labrum fused; six stemmata, clustered 4 + 2; spiracles uniforous with reticulate peritreme; egg bursters on TII and TIII and associated with a long and a short seta. Lamprosomatinae show a number of larval features in common with the cryptocephalines, being the body J-shaped among the most obvious (and related with their habit of carrying a case), as well as the fusion of frons, clypeus and labrum. Yet, unlike the cryptocephaline larvae, those of Lamprosomatinae have bicameral spiracles with peritreme simple, and five stemmata grouped 2 + 3. The maxillary palp 3-segmented plus the palpiger, as present in both groups, is likely a plesiomorphy, and although both subfamilies have egg-bursters confined to the meso- and metathorax, those in Lamprosomatinae lack the short ventral seta (Agrain and Marvaldi 2009, and references therein).

#### Tribe Clytrini Kirby, 1837

##### Subtribe Arateina Lacordaire, 1848

**Diagnosis.** Same as for genus, see below.

###### Aratea

Taxon classificationAnimaliaColeopteraChrysomelidae

Lacordaire, 1848

Fig. 4

Lacordaire 1848: 467; Chapuis 1874: 151; Jacoby and Clavareau 1906: 73; Guérin 1943: 86; Monrós 1953a: 261; Moldenke 1981: 88.

####### Type species.

*Aratea costata* Lacordaire, 1848. By monotypy.

**Figure 4.**
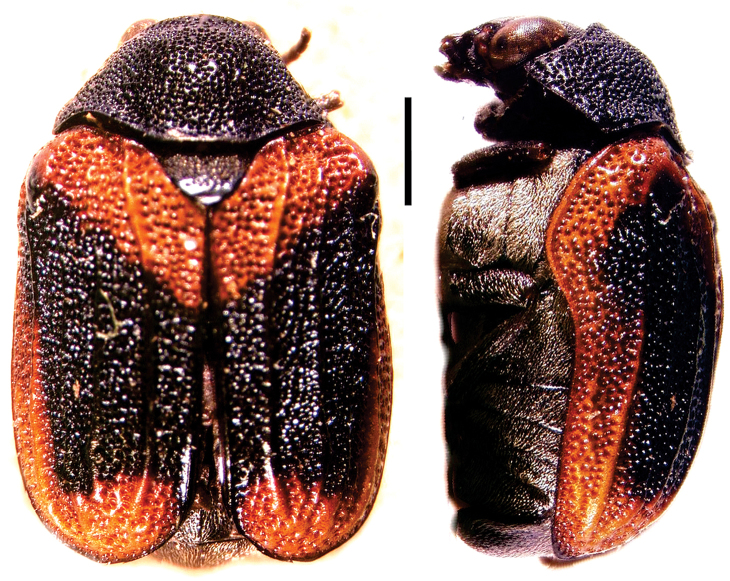
*Aratea costata* Lacordaire ^(1)^, left: habitus (dorsal view), right: habitus (lateral view).

####### Diagnosis.

This genus is easily recognized by the presence of strong parallel carinae on the elytra; other diagnostic characters include: frons very narrow; scutellum slanting posterodorsally, distinctly protruding from the plane of elytra; intercoxal prosternal process nearly absent between fore coxae; epipleural margin broadly angled, tip rounded; elytra without pubescence; frons with medial pit, densely and coarsely punctate throughout; and pygidium strongly convex.

####### Distribution.

Only two species from Brazil, one present in Argentina, likely to also occur in Paraguay.

####### Remarks.

Agrain and Roig-Juñent (2011), found eight autapomorphies for the genus, among them elytra with strongly marked longitudinal striae constitutes an exclusive synapomorphy to the genus.

####### Argentinian species checklist.

1. *Aratea costata* Lacordaire, 1848 (FOR, MNS, SEO).

##### Subtribe Babiina Chapuis, 1874

###### Babia

Taxon classificationAnimaliaColeopteraChrysomelidae

Chevrolat, 1836

Babia Chevrolat in Dejean 1836: 441, 1842: 409 in d’Orbigny; Lacordaire 1848: 424–425; Chapuis 1874: 147; Jacoby 1880: 33; Jacoby and Clavareau 1906: 70; Clavareau 1913: 81; Schaeffer 1933: 319–320; Guérin 1943: 65–66; Monrós 1953a: 212–213, 1953b: 46; Moldenke 1970: 132, 1981: 103.
=Harpasta Gistel, 1848: 123.

####### Note.

Moldenke (1981), divided *Babia* into six subgenera based on morphological features, such as the general shape of the body, pronotal margin, frons and antennomere morphology: Babia (Babia) Chevrolat and B. (Archaebabia) Moldenke from America north of Mexico; Babia (Coleolacordairei) Moldenke, Babia (Heterobabia) Moldenke, and Babia (Megababia) Moldenke from the Neotropical region. Only the monotypic B. (Coleolacordairei) is represented in Argentina.

###### Babia (Coleolacordairei)

Taxon classificationAnimaliaColeopteraChrysomelidae

Moldenke, 1981

Fig. 5

####### Type species.

*Babia elongata* Guérin, 1945. By monotypy.

**Figure 5.**
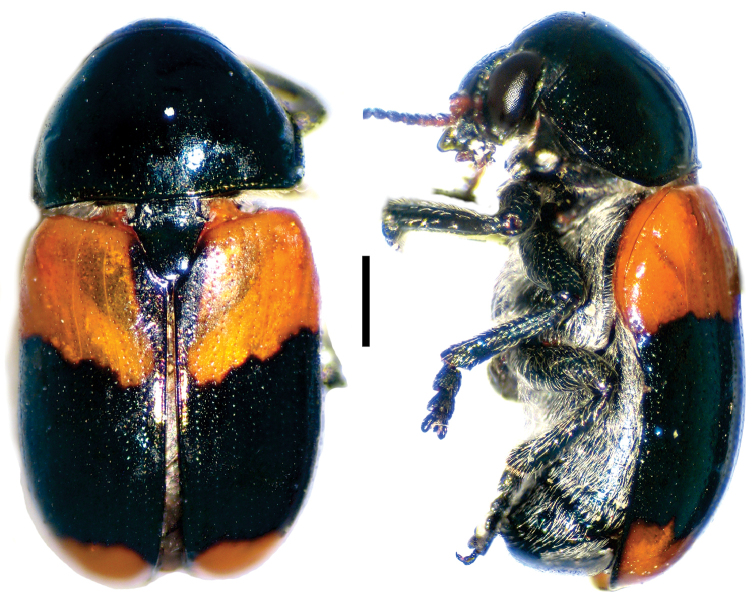
Babia (Coleolacordairei) elongata Guérin ^(1)^, left: habitus (dorsal view), right: habitus (lateral view).

####### Diagnosis.

This subgenus can be reliably diagnosed by the shape of anterior margin of pronotum, which is arcuate, and covers the entire head in dorsal view. Also, body shape is elongate (length 3x width), cylindrical and flat; frons is flat; lateral margin of prothorax not widely explanate.

####### Distribution.

Brazil and Argentina.

####### Remarks.

Moldenke (1981), mentioned the size of this species to be greater than 10 mm, but average size is smaller than 10 mm.

####### Argentinian species checklist.

1. Babia (Coleolacordairei) elongata Guérin, 1945 (BAS, COR, ERS, JUY, LRA, RNO, SAL, SFE, TUC). Host plant: Fabaceae: *Acacia* sp. (Monrós 1953a).

###### Cylindrodachrys

Taxon classificationAnimaliaColeopteraChrysomelidae

Monrós, 1944

Fig. 6

Monrós (1944: 148, 1953b: 148).

####### Type species.

*Cylindrodachrys cleroides* Monrós (1944: 148).

**Figure 6.**
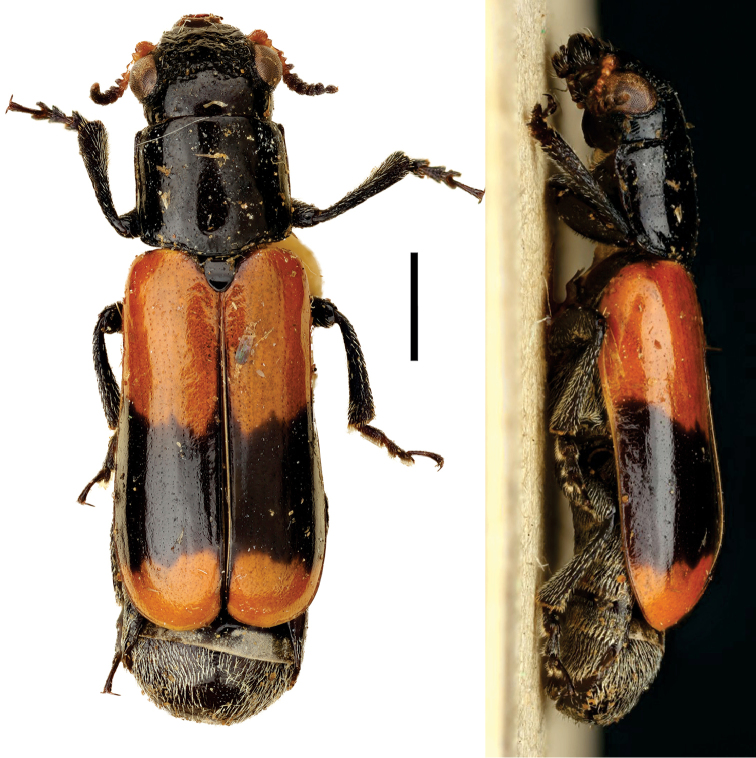
*Cylindrodachrys cleroides* Monrós ^(2)^, left: habitus (dorsal view), right: habitus (lateral view).

####### Diagnosis.

This genus exhibits a particular combination of three characters unique among Clytrini: adult body shape strongly cylindrically elongate; elytra not fully covering pygidium; and inconspicuous elytral punctations, thus superficially resembling a clerid.

####### Distribution.

This monotypic genus is limited to Paraguay and north and central Argentina.

####### Remarks.

According to Monrós (1944) this species has always been collected in extremely xeric places.

####### Argentinian species checklist.

*Cylindrodachrys cleroides* Monrós, 1944 (CAT, COR, MZA, SEO, SLS). Host plant: Solanaceae: *Solanum eleagnifolia* (Quillo); Malvaceae: *Gossypium hirsutum* (Monrós, 1953a).

###### Dachrys

Taxon classificationAnimaliaColeopteraChrysomelidae

Erichson, 1847

Fig. 7

Erichson 1847: 164; Lacordaire 1848: 405–406; Chapuis 1874: 146; Jacoby and Clavareau 1906: 68–69; Clavareau 1913: 80; Guérin 1943: 53–54; Monrós 1953a: 208–209, 1953b: 48–49; Moldenke 1970: 108.

####### Type species.

*Dachrys succincta* (Erichson, 1834), designated by Monrós (1953b: 48).

**Figure 7.**
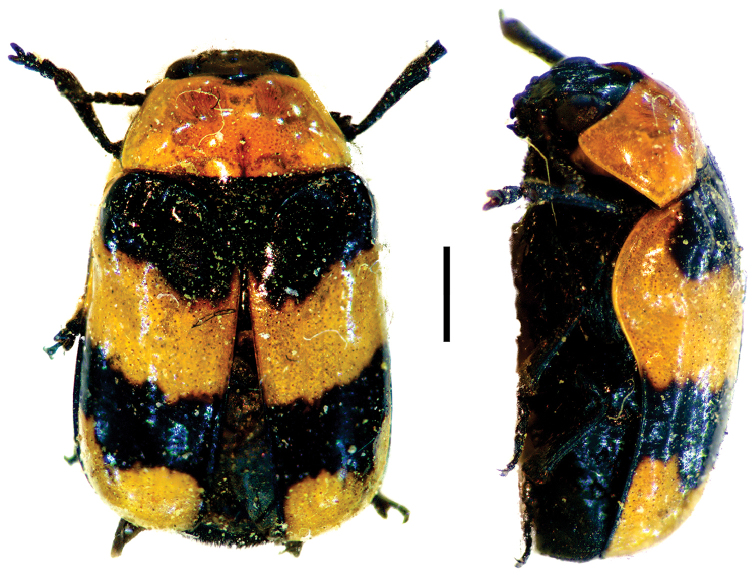
*Dachrys succincta* (Erichson) ^(1)^, left: habitus (dorsal view), right: habitus (lateral view).

####### Diagnosis.

This genus resembles *Saxinis*, but is distinguished by the epipleural lobe not well developed and from *Temnodachrys* by the sides of the prothorax curved (strongly convergent towards the head); frons with deep medial pit; and distinct elytral pattern with transverse black bands on reddish base color.

####### Distribution.

Austral region of Chile and Argentina.

####### Remarks.

Monrós (1953b) separated the genera: *Saxinodachrys*, *Cylindrodachrys*, and *Temnodachrys*, formerly considered within *Dachrys*. Currently monotypic.

####### Argentinian species checklist.

*Dachrys succincta* (Erichson, 1834) (CHU, NQN, RNO). Host plant: Rhamnaceae: *Discaria* sp. (Monrós, 1953a) and *Chacaya trimereus* (Roig-Juñent, 2004).

= *Clythra succincta* Erichson, 1834.

= *Dachrys succincta* Lacordaire, 1848.

###### Dinophthalma

Taxon classificationAnimaliaColeopteraChrysomelidae

Lacordaire, 1848

Fig. 8

Dinophthalma Lacordaire 1848: 400; Chapuis 1874: 145; Jacoby and Clavareau 1906: 67; Guérin 1943: 47; Monrós 1953a: 143.

####### Type species.

*Dinophthalma ophthalmica* Lacordaire, 1848 designated by Monrós (1953a: 143).

**Figure 8.**
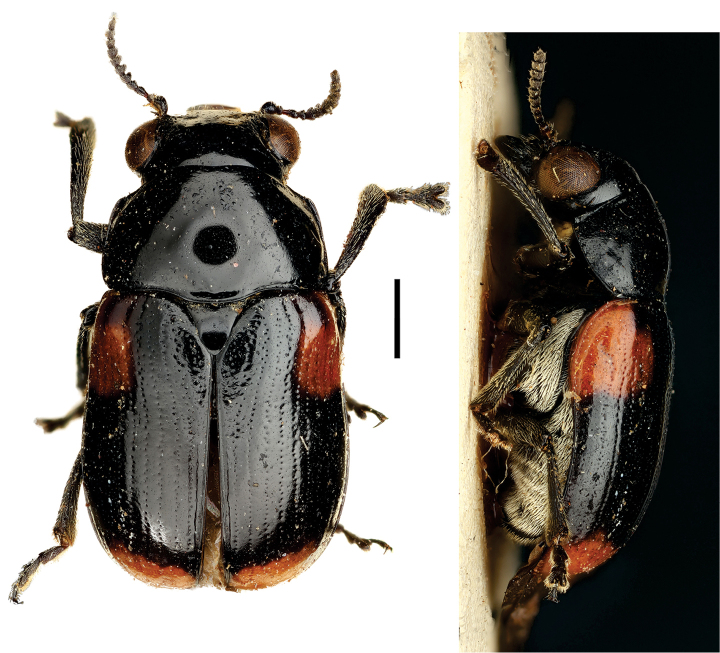
*Dinophthalma amplicollis* Monrós ^(2)^, left: habitus (dorsal view), right: habitus (lateral view).

####### Diagnosis.

Small body size (less than 7 mm). This genus is very similar to *Temnodachrys*, from which it can be easily separated by the extraordinary development of the eyes, which are protruding and reaching the lateral margins of the head. Also, antennae with antennomere III large, conical; pronotum transverse; elytra without callus.

####### Distribution.

Brazil, Ecuador, Bolivia, Paraguay, and Argentina.

####### Argentinian species checklist.

*Dinophthalma amplicollis* Monrós, 1953 (MNS).

*Dinophthalma discicollis consimilis* Baly, 1877a (FOR, MNS).

= *Dinophthalma consimilis* Baly, 1877a.

= *Babia pallidipennis* Guérin, 1943.

###### Helioscopa

Taxon classificationAnimaliaColeopteraChrysomelidae

Gistel, 1848

Fig. 9

Helioscopa Gistel: 1848: 123, Monrós and Bechyné 1956: 1122.
=Acidalia Chevrolat, 1836
=Tellena Lacordaire, 1848
=Tellenina Monrós, 1953a.

####### Type species.

*Clythra varians* Sahlberg, 1823. By monotypy.

**Figure 9.**
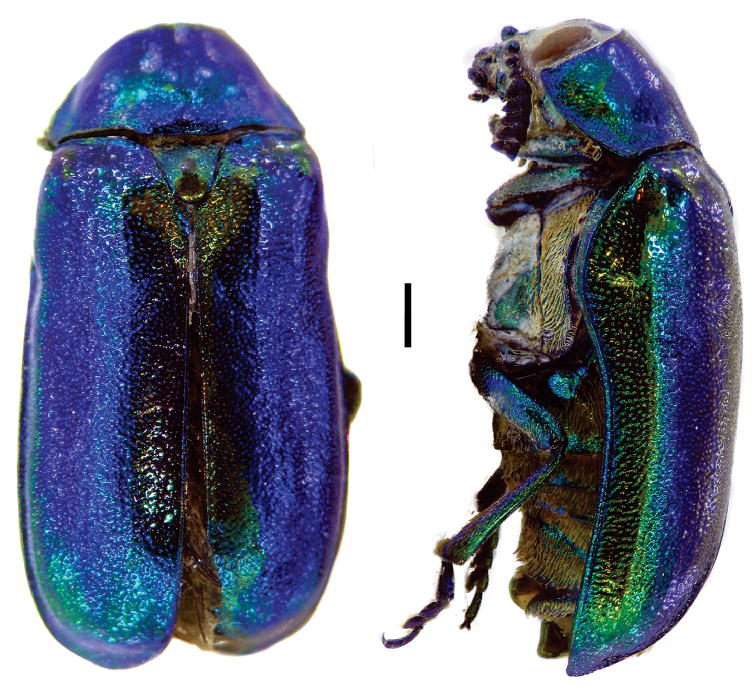
*Helioscopa varians varians* (Sahlberg) ^(1)^, left: habitus (dorsal view), right: habitus (lateral view).

####### Diagnosis.

body elongate, brilliant metallic, uniform green/blue coloration; and bifid tarsal claws. Also, antennae serrated from IV antennomere; pronotal margin slightly marginate; scutellum long, triangular, with round apex; legs long, with long tarsi, last tarsomere surpassing ½ the lobes of tarsomere III.

####### Distribution.

Southern Brazil, Argentina and Paraguay. Only two subspecies of this genus have been cited in Argentina.

####### Argentinian species checklist.

1a. *Helioscopa varians varians* (Sahlberg, 1823) (MNS).

= *Clythra varians* Sahlberg, 1823.

= *Tellena varians* Lacordaire, 1848.

1b. *Helioscopa varians angusticollis* (Jacoby, 1897) (CHA, COR, JUY, MNS, SEO, TUC). Host plant: Boraginaceae: *Cordia salviflora*, Argentina / Monrós, (1953a).

= *Tellena angusticollis* Jacoby, 1897.

###### Paraurodera

Taxon classificationAnimaliaColeopteraChrysomelidae

Moldenke, 1981

Fig. 10

Paraurodera Moldenke (1981: 110).

####### Note.

This genus was created by Andrew Moldenke (1981) to include seven species previously included in *Urodera*. Moldenke (1981) divided it into two subgenera as follows:

**Figure 10.**
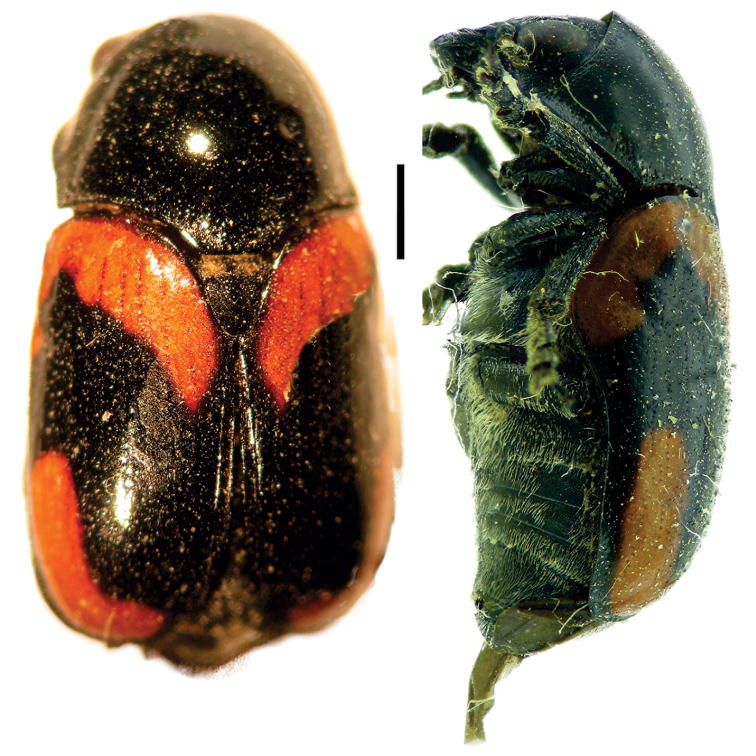
Paraurodera (Paraurodera) haematifera (Lacordaire) ^(1)^, left: habitus (dorsal view), right: habitus (lateral view).

###### Paraurodera (Paraurodera)

Taxon classificationAnimaliaColeopteraChrysomelidae

Moldenke, 1981

####### Type species.

*Paraurodera similis* Moldenke, 1981, by original designation.

####### Diagnosis.

According to Moldenke (1981), this group can be separated from *Urodera* by the following combination of characters: subrectangular body shape, with sides subparallel; anterior margin of pronotum never concealing entire head in dorsal view; legs without longitudinal carinae. Additional characters that may help distinguish this genus are: frons without strong sexual dimorphism; antennomere IV clavate, smaller than V; hind pronotal angles sinuate, perpendicular.

####### Distribution.

Argentina, Brazil, Colombia, and Uruguay.

####### Argentinian species checklist.

Paraurodera (Paraurodera) bergi (Harold, 1875) (CHA, COR, CTS, LRA, MNS, MZA, SEO, SFE). Host plant: Fabaceae: *Acacia caven* (Mol.) Mol. (Viana and Williner 1972).

= *Stereoma bergi* Clavareau, 1913.

Paraurodera (Paraurodera) hamatifera densepunctata Monrós, 1953a (CHA, COR, FOR, JUY, MNS, SAL).

Paraurodera (Paraurodera) hamatifera hamatifera (Lacordaire, 1848) (CHA, COR, CTS, ERS, FOR, LRA, MNS, MZA, SAL, SEO, SFE, SJN, SLS, TUC). Host plant: Fabaceae: *Prosopis* sp. (Monrós, 1953a), *Prosopis nigra* (Ward et. al., 1977) and *Prosopis alpataco* (Roig-Juñent, 2004); *Acacia farnesiana* (Viana and Williner, 1974); *Hieronimus* sp. (Ward et al., 1977).

Paraurodera (Paraurodera) inornata (Monrós, 1953a) (CHA).

Paraurodera (Paraurodera) similis Moldenke, 1981 (JUY, SAL, FOR, CHA, MNS, SEO, TUC, CTS, COR, CAT, SFE, BAS, MZA). Host plant: Zygophyllaceae: *Zucagnia punctata* flowers (Roig-Juñent, 2004).

= *Urodera vau* Monrós, 1953a (nec Lacordaire, 1848).

###### Paraurodera (Torourodera)

Taxon classificationAnimaliaColeopteraChrysomelidae

Moldenke, 1981

####### Type species.

*Urodera fallax* Harold, 1875, designated subsequently by Moldenke (1981: 111).

####### Diagnosis.

Anterior margin of pronotum not explanate; male head very conspicuous; frons with very strong sexual dimorphism, male mandibles prominent; antennomere IV much smaller than V; hind pronotal angles obtuse.

####### Distribution.

This subgenus is endemic to Argentina.

####### Argentinian species checklist.

Paraurodera (Torourodera) duplicata (Monrós, 1953a) (CAT, CHA, COR, CTS, FOR, MNS, SEO, SFE, SLS). Host plant: Fabaceae: *Prosopis* sp. and *P. rucifolia* (Ward et al., 1977), *Prosopis alba* (Viana and Williner, 1974).

Paraurodera (Torourodera) fallaciosa (Monrós, 1953a) (COR, CTS, SFE, SLS).

Paraurodera (Torourodera) fallax (Harold, 1875) (BAS, CAT, CHA, COR, CTS, ERS, FOR, JUY, MNS, MZA, SAL, SEO, SFE, TUC). Host plants: Fabaceae: *Sesbania punicea*, and *S. virgata* (Monrós, 1953a); *Prosopis caldenia* (Aravena, 1940; 1974).

###### Pnesthes

Taxon classificationAnimaliaColeopteraChrysomelidae

Lacordaire, 1848

Fig. 11

Lacordaire (1848: 403); Chapuis (1874: 1–16); Jacoby and Clavareau (1906: 68); Guérin (1943: 51).

####### Type species.

*Pnesthes ligata* Lacordaire designated by Monrós (1953a: 150).

**Figure 11.**
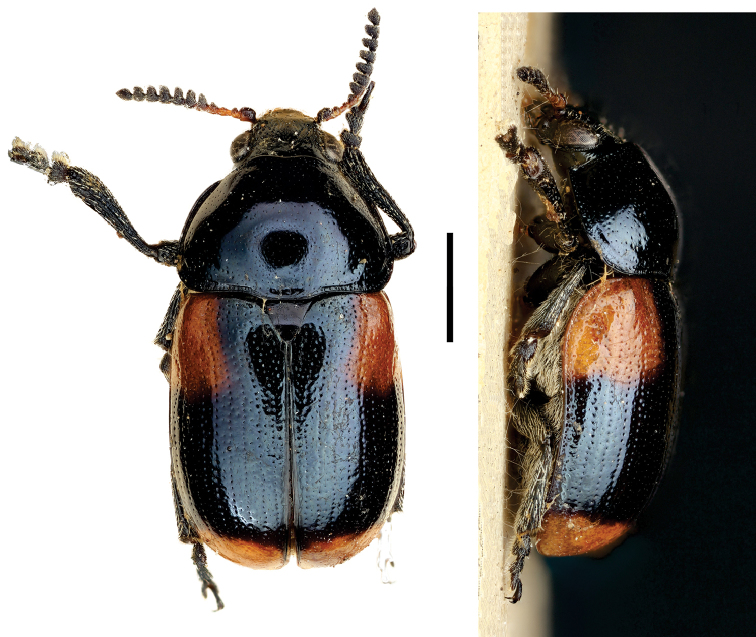
*Pnesthes instabilis minuta* Monrós ^(2)^, left: habitus (dorsal view), right: habitus (lateral view).

####### Diagnosis.

The most particular character to diagnose this genus is the shape of the head, which is anteriorly prolonged, strongly tapering and triangular. Other diagnostic characters are: elongate body, shiny and glabrous; eyes moderately salient; pronotal base lobate; scutellum long and,triangular.

####### Distribution.

Two species from Brazil, one of these with a subspecies in Northeastern Argentina which was separated from the typical form by Monrós (1953a) on the basis of its smaller size and distinct punctuation.

####### Argentinian species checklist.

*Pnesthes instabilis minuta* Monrós, 1953a (MNS, SFE).

###### Stereoma

Taxon classificationAnimaliaColeopteraChrysomelidae

Lacordaire, 1848

Fig. 12

Stereoma Lacordaire 1848: 437; Chapuis 1874: 148; Jacoby 1880: 34; Jacoby and Clavareau 1907: 71; Guérin 1943: 72; Monrós 1953a: 215; Moldenke 1981: 107.

####### Type species.

*Stereoma clitellata* Lacordaire, designated by Monrós (1953a): 215.

**Figure 12.**
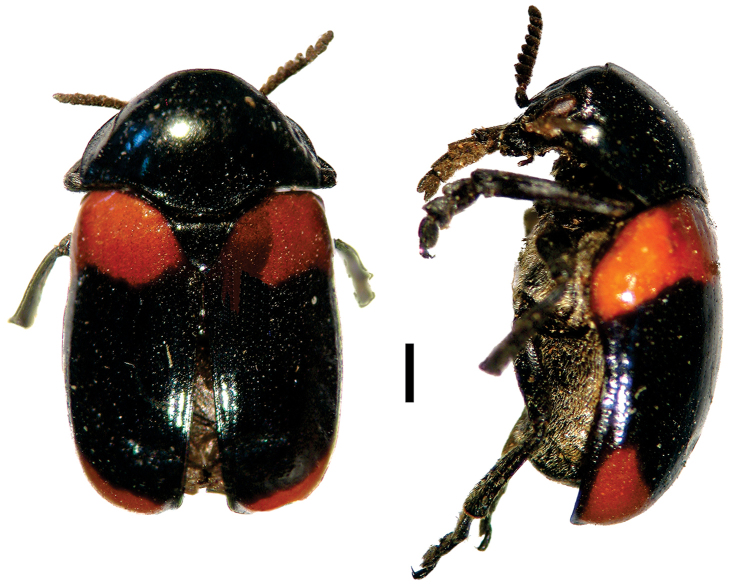
*Stereoma clitellata clitellata* Lacordaire ^(1)^, left: habitus (dorsal view), right: habitus (lateral view).

####### Diagnosis.

Sexually dimorphic, with males having enlarged forelegs; head completely concealed within prothorax; mesosternum broad; tarsal segments very transverse, tarsomere III cleft ^1^/_2_ length to receive IV; tarsomere IV notoriously short and thick; frons with prominent transverse sulcation; lateral margin of pronotum broadly explanate, hind angles broadly rounded. This genus is closely related to *Urodera*, from which it can be separated by the conspicuous development of the legs, since the latter is much larger in males and females compared to *Urodera*.

####### Distribution.

Seven species and four subspecies are known from Argentina, another 10 species are known from Meridional America (southern part of South America).

####### Remarks.

*Sesbania virgata* (Fabaceae) has been cited as a hostplant for *Stereoma* sp. in Argentina (Monrós 1953a).

####### Argentinian species checklist.

*Stereoma anchoralis* Lacordaire, 1848 (COR).

*Stereoma angularis* Lacordaire, 1848 (MNS).

3a. *Stereoma clitellata burmeisteri* Harold, 1875 (CHA, COR, FOR, JUY, LRA, MNS, SAL, SEO, SFE, TUC).

= *Stereoma burmeisteri* Harold, 1875.

3b. *Stereoma clitellata clitellata* Lacordaire, 1848 (CHA, CTS, FOR, MNS, SFE). Host plant: Fabaceae: *Acacia decurrens* (Monrós, 1953a).

3c. *Stereoma clitellata concolor* Lacordaire, 1848 (JUY, SAL, SEO).

= *Stereoma concolor* Lacordaire, 1848.

4a. *Stereoma laevicollis bosqui* Monrós, 1953a (SFE).

4b. *Stereoma laevicollis laevicollis* (Harold, 1875) (CAT, CHA, FOR, JUY, LRA, SAL, SEO, SLS). Host plant: Fabaceae: *Cassia aphylla* (Viana & Williner, 1974).

= *Urodera laevicollis* Harold, 1875.

4c. *Stereoma laevicollis orophila* Monrós, 1953a (JUY, SAL, TUC).

5. *Stereoma obesa* Monrós, 1953a (SEO).

6. *Stereoma seenoi* Moldenke, 1981 (JUY). [Moldenke (1981), mistakenly cited this species from (Jujuy: Bolivia), it belongs to Jujuy: Argentina.].

###### Saxinis

Taxon classificationAnimaliaColeopteraChrysomelidae

Lacordaire, 1848

Saxinis Lacordaire 1848: 478–79; Chapuis 1874: 150; Jacoby 1880: 36–37; Horn 1892: 8; Jacoby and Clavareau 1906: 74; Clavareau 1913: 84; Guérin 1943: 88; Blackwelder 1946: 639; Monrós 1953a: 257–258; Moldenke 1970: 154, 1981: 106.

####### Note.

Moldenke (1981) erected Saxinis (Boreosaxinis) to include North American species. The other four species of Saxinis are included in the nominotypic subgenus and distributed in Central and South America, with only one species described from Argentina.

###### Saxinis (Saxinis)

Taxon classificationAnimaliaColeopteraChrysomelidae

Lacordaire, 1848

Fig. 13

Moldenke 1981: 106.

####### Type species.

*Saxinis sagittaria* Lacordaire, 1848, designated by Monrós (1953a: 257).

**Figure 13.**
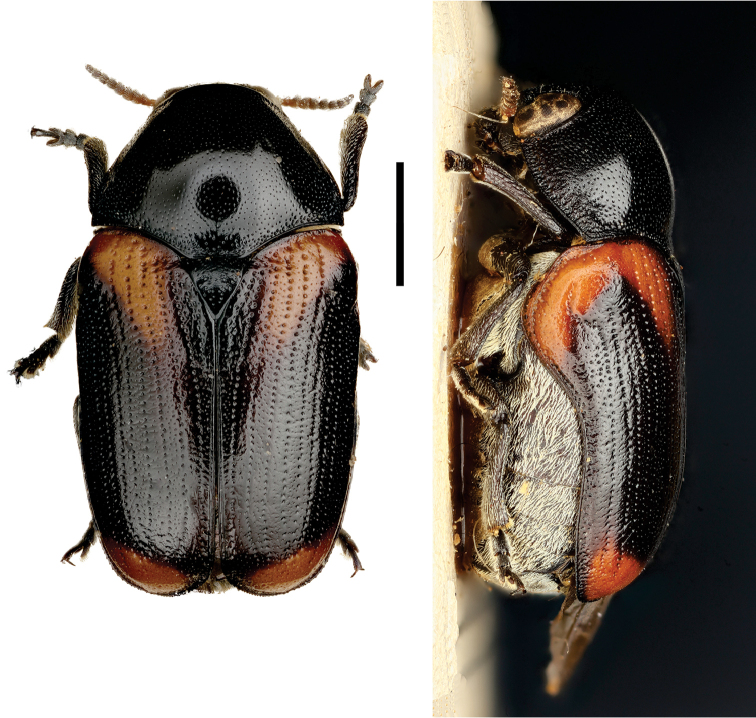
Saxinis (Saxinis) meridionalis Monrós ^(2)^, left: habitus (dorsal view), right: habitus (lateral view).

####### Diagnosis.

This group can be easily distinguished from all other members of this subtribe by the very large and generally pointed epipleurae. South American species exhibit a black dorsal coloration always with metallic bronze reflections.

####### Distribution.

Usually foundin Central and South America.

####### Argentinian species checklist.

Saxinis (Saxinis) meridionalis Monrós, 1953a (BAS, CHA, COR, ERS, FOR, JUY, LPA, LRA, MZA, SAL, TUC).

###### Temnodachrys

Taxon classificationAnimaliaColeopteraChrysomelidae

Monrós, 1953

Fig. 14

Dachrys Lacordaire, 1848 (part)
Temnodachrys Monrós 1953a: 153–15, 1953b: 48–49; Moldenke 1970: 109, 1981: 107.

####### Note.

Monrós (1953b) divided this diverse genus (more than 60 species) into two subgenera based on the presence of a deep transverse sulcus in the interocular region.

**Figure 14.**
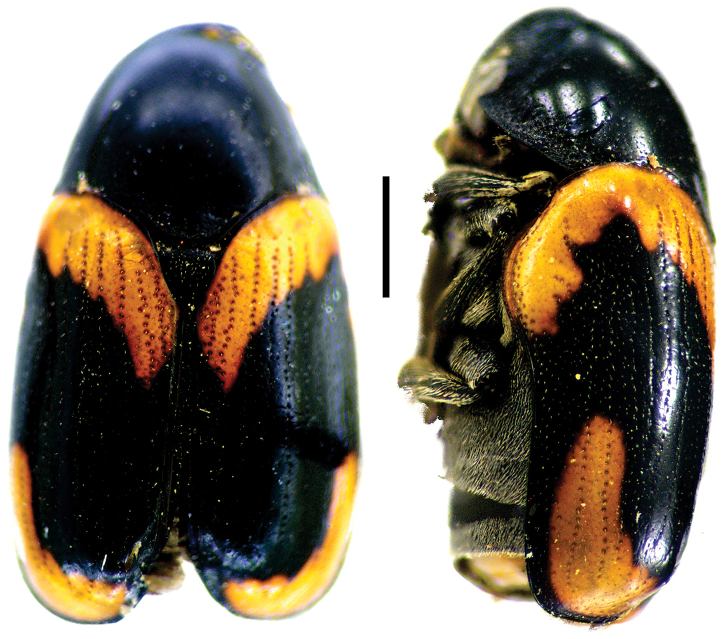
Temnodachrys (Temnodachrys) aeneofasciata (Lacordaire) ^(1)^, left: habitus (dorsal view), right: habitus (lateral view).

###### Temnodachrys (Eudachrys)

Taxon classificationAnimaliaColeopteraChrysomelidae

Monrós, 1953

####### Type species.

*Temnodachrys cruciata* (Lacordaire, 1848), designated by Monrós (1953b: 49).

####### Diagnosis.

Frons without deep transverse sulcus; body minute, drop-like shaped with sides subparallel.

####### Distribution.

This subgenus has over 60 species and is distributed from Mexico to Argentina and Chile, 22 species have been cited for Argentina.

####### Argentinian species checklist.

1a. Temnodachrys (Eudachrys) complexa complexa (Lacordaire, 1848) (BAS, CHA, CTS, ERS, MNS, SEO, SFE, TUC). Host plant: Rosaceae: *Rosa* sp. (Roses) (Monrós 1953a).

1b. Temnodachrys (Eudachrys) complexa pallipes Monrós, 1953a (BAS, CTS, FOR, MNS, SFE).

Temnodachrys (Eudachrys) cruciata (Lacordaire, 1848) (BAS, COR, MNS, MZA, SEO, SFE). Host plant: Asteraceae: *Eryngium* sp. (Monrós, 1953a).

Temnodachrys (Eudachrys) decolorata Monrós, 1953a (SEO).

Temnodachrys (Eudachrys) haywardi Monrós, 1953a (NQN).

Temnodachrys (Eudachrys) impressifrons Monrós, 1953a (SFE).

Temnodachrys (Eudachrys) lacordairei Monrós, 1953a (JUY, SAL).

Temnodachrys (Eudachrys) laeta (Lacordaire, 1848) (JUY, MNS).

Temnodachrys (Eudachrys) longipennis (Guérin, 1943) (FOR, MZA).

Temnodachrys (Eudachrys) manca (Harold, 1875) (COR, MZA, SEO).

= *Urodera manca* Clavareau, 1913.

Temnodachrys (Eudachrys) monticola Monrós, 1953a (TUC).

Temnodachrys (Eudachrys) oyaguava Monrós, 1953a (COR, MNS).

Temnodachrys (Eudachrys) punctipennis (Monrós, 1951b) (MNS).

Temnodachrys (Eudachrys) puntana Monrós, 1953a (COR, MZA, SLS). Host plant: Fabaceae: *Cercidium praecox* (Ruiz & Pavon ex Hook.) Harms; (Roig-Juñent 2004).

Temnodachrys (Eudachrys) pygmaea Monrós, 1953a (JUY).

Temnodachrys (Eudachrys) sympathica Monrós, 1953a (CHA, COR, SEO).

Temnodachrys (Eudachrys) taeniatoides Monrós, 1953a (MNS).

Temnodachrys (Eudachrys) trisignata (Lacordaire, 1848) (COR, JUY, SEO, TUC).

Temnodachrys (Eudachrys) trivirgata (Lacordaire, 1848) (CAT, SAL). Host plant: Astaeraceae (Monrós 1947).

Temnodachrys (Eudachrys) vianai Monrós, 1953a (MNS).

Temnodachrys (Eudachrys) willinki Monrós, 1953a (CHA, COR, FOR, MNS, MZA, SAL, SEO, SFE, TUC). Host plant: Fabaceae: *Prosopis* sp. (Monrós, 1953a).

Temnodachrys (Eudachrys) wygodzinskyi Monrós, 1953a (JUY).

Temnodachrys (Eudachrys) xerophila Monrós, 1953a (FOR, SEO, LRA, COR, SLS). Host plant: Solanaceae: *Solanum eleagnifolia* Cav. (Quillo) (Monrós, 1953a).

###### Temnodachrys (Temnodachrys)

Taxon classificationAnimaliaColeopteraChrysomelidae

Monrós, 1953

####### Type species.

*Temnodachrys aeneofasciata* (Lacordaire, 1848), designated by Monrós (1953b: 49).

####### Diagnosis.

Frons with deep transverse sulcus; body shape subrectangular.

####### Distribution.

Seven species, from northern Brazil (one species) to central Argentina (six species).

####### Remarks.

The characters mentioned by Guérin (1952) in the original description of *Dachrys argentina*, are sufficient to transfer this species to this subgenus. The author mentioned the presence of a deep transverse sulcus in interocular region. Secondly, sides of the prothorax are much less convergent that expected and pronotal and elytral coloration pattern does not fit *Dachrys*. In addition, distribution of *Dachrys* is limited to Chile and southern Argentina, while Jujuy is the northernmost province. Finally, Guérin indicated the similarity with T. (T.) signatipennis (Lacordaire), and *Dachrys gracilis* Harold [=T. (T.) aeneofasciata (Lacordaire)].

####### Argentinian species checklist.

Temnodachrys (Temnodachrys) argentina (Guérin, 1952), **comb n.** (JUY).

= *Dachrys argentina* Guérin, 1952.

Temnodachrys (Temnodachrys) aeneofasciata (Lacordaire, 1848) (BAS, CHA, COR, CTS, ERS, FOR, JUY, LPA, MNS, MZA, NQN, SAL, SEO, SFE, TUC). Host plant: Fabaceae: *Sesbania marginata*, *Sesbania virgata*, and *Prosopis algarrobilla* (Monrós 1953a); *Prosopis caldenia* (Aravena 1974); *Prosopis affinis*. (Monrós 1953a).

= *Dachrys gracilis* Harold, 1875.

= *Dachrys aeneofasciata* Lacordaire, 1848.

Temnodachrys (Temnodachrys) aphodiodes (Lacordaire, 1848) (BAS, COR, ERS, LPA, MNS, SFE). Host plant: Fabaceae: *Mimosa farinosa* (Monrós 1953a).

Temnodachrys (Temnodachrys) hybrida Monrós, 1953a (ERS).

Temnodachrys (Temnodachrys) neffi Moldenke, 1981 (CAT). Host plant: Fabaceae: *Prosopis torquata* (Cavanilles ex Lagasca) D.C., *Prosopis chilensis* (Molina) Stuntz emend.; *Mimosa farinose* Griseb, *Mimosa ephedroides* (Gillies ex Hook. & Arn.) Benth.

Temnodachrys (Temnodachrys) pauperrima Monrós, 1953a (JUY, SAL, CHA, LRA).

Temnodachrys (Temnodachrys) quichua Monrós, 1953a (JUY, SAL).

Temnodachrys (Temnodachrys) signatipennis (Lacordaire, 1848) (JUY, SAL, FOR, MNS, TUC, SEO, CAT, COR, LRA, CTS, SFE, SLS, BAS). Host plants: Fabaceae: *Acacia caven* (Mol.) Mol. (Monrós 1953a); *Piptadenia macrocarpa* Benth and *Piptadenia cebil* (Griseb.) (Jolivet, 1978); *Sesbania virgata* (Cav.) Argentina / (Monrós 1953a); *Anadenanthera colubrina var cebil* (Vell. Conc.) Brenan Argentina / (Jolivet 1978; Hayward 1944).

###### Urodera

Taxon classificationAnimaliaColeopteraChrysomelidae

Lacordaire, 1848

Fig. 15

Urodera Lacordaire 1848: 449; Chapuis 1874: 149; Jacoby 1880: 34–35; Jacoby and Clavareau 1906: 72; Clavareau 1913: 83; Leng 1920: 288; Guérin 1943: 80–81; Blackwelder 1946: 638; Monrós 1953a: 232–233; Moldenke 1970: 114, 1981: 112.

####### Note.

Moldenke (1981) divided this genus into five subgenera (including the nominotypic one). Two of these subgenera are present in Argentina, plus two species regarded by Moldenke (1981) as *incertae sedis*.

**Figure 15.**
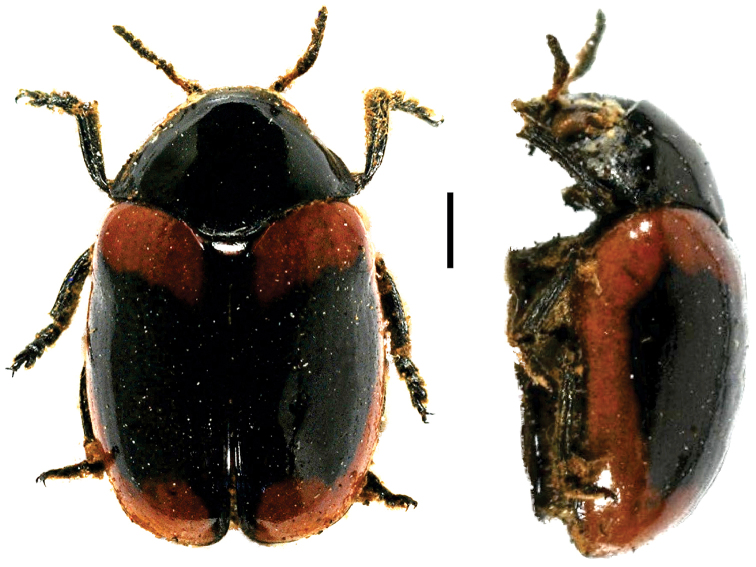
Urodera (Austrurodera) circumcincta circumcincta Lacordaire ^(3)^, left: habitus (dorsal view), right: habitus (lateral view).

###### Urodera (Austrurodera)

Taxon classificationAnimaliaColeopteraChrysomelidae

Moldenke, 1981

Urodera (Austrurodera) Moldenke 1981: 113.

####### Type species.

*Urodera quadrisignata* Lacordaire, 1848, designated by Moldenke (1981: 113).

####### Diagnosis.

Posterior margin of pronotum broadly expanded, forming distinct scutellar lobe usually bounded by acute angles; scutellum posteriad humeral callus, scutellum nearly as long as length of posterior lobe of pronotum; aedeagus with broad weakly-sclerotized dorsal medial flap.

####### Distribution.

Argentina, Brazil, Colombia, Costa Rica, Guatemala, Panama, Paraguay, and Venezuela.

####### Remarks.

Moldenke (1981) subdivided this subgenus into two groups. Argentinian species belong to type II group, which are characterized by having protibiae twice carinate on posterolateral surface; and antennomere IV ^1^/_3_ - ^3^/_4_ times width of V.

####### Argentinian species checklist.

1a. Urodera (Austrurodera) circumcincta circumcincta Lacordaire, 1848 (MNS, CTS, CHA, JUY).

1b. Urodera (Austrurodera) circumcincta circumducta Lacordaire, 1848 (MNS, CTS, CHA).

= *Urodera circumducta* Lacordaire, 1848

2. Urodera (Austrurodera) monrosi Moldenke, 1981 (MNS).

###### Urodera (Stereomoides)

Taxon classificationAnimaliaColeopteraChrysomelidae

Moldenke, 1981

Moldenke 1981: 114.

####### Type species.

*Stereoma tetraspilota* Lacordaire, 1848, designated by Moldenke (1981: 113).

####### Diagnosis.

Scutellum length 1.5x or more than length of posterior lobe of pronotum; frons of male with deep medial depression; dorsal region of aedeagus with very prominent row of setae, no cleft, ventral lobe absent, apex of aedeagus extraordinarily truncate.

####### Distribution.

Argentina, Bolivia, Brazil, Paraguay, and Peru.

####### Argentinian species checklist.

Urodera (Stereomoides) neffi Moldenke, 1981 (CAT). Host plant: Asteraceae: *Baccharis* sp.

Urodera (Stereomoides) tetraspilota (Lacordaire, 1848) (MNS, SAL).

= *Stereoma tetraspilota* Lacordaire, 1848.

##### *Urodera* incertae cedis by Moldenke (1981)

1. *Urodera lanuginosa* Monrós, 1953a (SEO, SFE).

2. *Urodera crucifera crucifera* Lacordaire, 1848 (*sensu* Monrós) (JUY, SAL, TUC).

= *Urodera hoepfneri* Lacordaire, 1848.

= *Urodera chevrolati* Lacordaire, 1848.

##### Subtribe Ischiopachina Chapuis, 1874

As mentioned above, further studies are necessary to clarify the relationships of Arateina and Ischiopachina with the remaining subtribes.

###### Ischiopachys

Taxon classificationAnimaliaColeopteraChrysomelidae

Chevrolat, 1836

Fig. 16

Ischiopachys Chevrolat in Dejean 1836: 440; Lacordaire 1848: 468–469; Chapuis 1874: 153; Jacoby 1880: 37; Jacoby and Clavareau 1906: 75–76; Clavareau 1913: 85; Guérin 1943: 90; Blackwelder 1946: 639; Monrós 1953a: 263–264, 1953b: 46; Moldenke 1970: 190.

####### Type species.

*Ischiopachys bicolor* (Olivier, 1791), designated by Monrós (1953b: 46).

**Figure 16.**
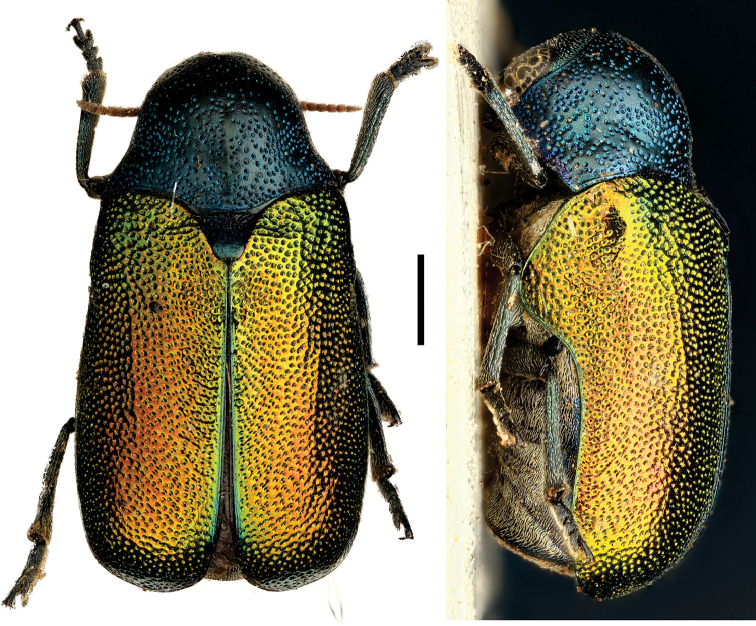
*Ischiopachys cribipennis cribipennis* Lacordaire ^(2)^, left: habitus (dorsal view), right: habitus (lateral view).

####### Diagnosis.

This genus has several diagnostic characters that clearly separate it from all other Neotropical Clytrini: scutellum inclined posterodorsally, distinctly protruding from the plane of elytra; intercoxal prosternal process nearly absent between fore coxae; epipleural margin broadly angled, tip rounded; elytra without pubescence; frons with medial pit, densely and coarsely punctate throughout; pygidium strongly convex; pronotum with lateral margins sulcate to receive antennae in repose; dorsum brilliant metallic.

####### Distribution.

From Mexico to Argentina.

####### Argentinian species checklist.

1a. *Ischiopachys cribipennis cribipennis* Lacordaire, 1848 (CTS). Host plant: *Sfolocalyx*? and *curupatí* (Monrós, 1953a).

1b. *Ischiopachys cribipennis micans* Lacordaire, 1848 (CHA, CTS, ERS, FOR, JUY, MNS, SAL, SEO, SFE, TUC). Host plants: Fabaceae: *Prosopis* sp.; *Piptadenia* sp.; *Caesalpinia* sp.sp. (Monrós, 1953a). Rutaceae: *Citrus* sp. (Naranjo) (Monrós, 1953a).

*Ischiopachys micans* Lacordaire, 1848.

2. *Ischiopachys empyrea empyrea* Lacordaire, 1848 (TUC, SAL).

= *Ischiopachys empyrea smaragdina* Monrós, 1953a.

##### Subtribe Megalostomina Chapuis, 1874

Major classification changes in Megalostomina were implemented based on the development of sexual dimorphic characters, especially as they relate to head modifications in males (Agrain and Roig-Juñent 2011). The monophyly of the subtribe is well supported by a set of synapomorphies, including external morphology and genitalia (Agrain and Roig-Juñent 2011).

###### Coscinoptera

Taxon classificationAnimaliaColeopteraChrysomelidae

Lacordaire, 1848

Fig. 17

Coscinoptera Lacordaire 1848: 511; Chapuis 1874: 139; Jacoby 1880: 31; Horn 1892: 12–13; Jacoby and Clavareau 1906: 62–63; Clavareau 1913: 77; Leng 1920: 288; Guérin 1943: 32–33; Blackwelder 1946: 637; Monrós 1951a: 1150–1151; Monrós 1953a: 114–115; Moldenke 1970: 41, 1981: 89.

####### Type species.

*Coscinoptera desmiphora* Lacordaire, 1848, designated by Monrós (1953a: 114).

**Figure 17.**
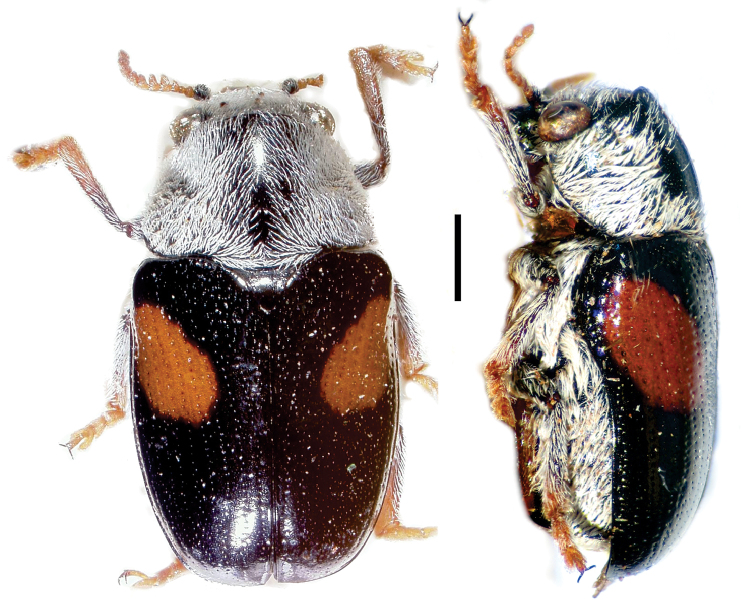
*Coscinopteraalbopilosa* (Monrós) ^(1)^, left: habitus (dorsal view), right: habitus (lateral view).

####### Diagnosis.

This genus can be separated from *Euryscopa* by the lack of bilobed lacinia and the elytra with confused punctation, in some cases exceedingly coarsely and deeply punctate. Other useful diagnostic characters are: head moderately prominent, covered with dense fine punctation and silky pubescence; male head as long as wide; eyes round and salient; prothorax transverse, pronotal disc as high as long; scutellum often coarsely punctate and always with dense white pubescent; elytra either metallic unicolored and glabrous or black; ventrites usually covered with exceedingly dense white pubescence; female anal pit normally small and only moderately depressed.

####### Distribution.

USA to Argentina.

####### Remarks.

As for other groups within Clytrini, it is in need of modern taxonomic revision. Several species groups have been proposed, but their monophyly has not yet been tested. Monrós (1953a), split this genus into two informal species groups, mainly based on sexual dimorphism evident in male heads. Moldenke (1970) proposed six informal species groups. Subsequently, Moldenke (1981) preserved only three of his earlier groups and transferred some species to two new genera (*Coleorozena* and *Coleothorpa*). More recently, Agrain and Roig-Juñent (2011), recovered a monophyletic clade (sister to *Megalostomis*), containing the type species of *Coscinoptera*, *Coleorozena*, and *Coleothorpa*. This clade is supported by two synapomorphies: male head as long as wide, and pronotal disc as high as long. Consequently, the latter two genera were synonymized with *Coscinoptera*. Some North American species are found in the nests of ant genera *Camponotus* Mayr and *Formica* Linnaeus. Moldenke (1981) divided this genus into three species groups and he indicated three species to be present in Argentina: *Coscinoptera euryscopoides* Monrós, and *Coscinopteraterebellum* Lacordaire within group II; and *Coscinoptera tibialis* Harold within group III.

####### Argentinian species checklist.

1. *Coscinopteraalbopilosa* (Monrós, 1953a) (BAS, CHA, COR, ERS, MNS, SEO, SFE). Host plant: Asteraceae: *Baccharis* sp. (branches). Zygofilaceae: on flowers (Monrós, 1947).

= *Euryscopa scapularis* Guérin, 1945; nec Lacordaire, 1848.

*Euryscopa albopilosa* Monrós, 1953a.

*Coleorozena albopilosa* Moldenke, 1981.

2. *Coscinopteraargentina* Burmeister, 1877 (COR, ERS, SEO).

*Euroscopa (Coleoguerina) argentina*: Moldenke, 1981 (misspelled for *Euryscopa*).

3. *Coscinoptera atypica* Monrós, 1953a (MNS).

*Euroscopa (Coleoguerina) atypica*: Moldenke, 1981 (misspelled for *Euryscopa*).

4. *Coscinoptera denieri* Monrós, 1953a (CHA, FOR).

*Euroscopa (Coleoguerina) denieri*: Moldenke, 1981 (misspelled for *Euryscopa*).

5. *Coscinopteradubia* Guérin, 1949 (COR, MZA, SEO).

= *Coscinopteraargentina* Guérin, 1944, not Burmeister, 1877.

*Euroscopa (Coleoguerina) dubia*: Moldenke, 1981 (misspelled for *Euryscopa*).

6. *Coscinoptera euryscopoides* Monrós, 1953a (SAL, SEO).

7. *Coscinopteraguerini* (Monrós, 1953a) (CHA, CTS, FOR, MNS).

*Coleorozena guerini*: Moldenke, 1981.

*Euryscopa guerini* Monrós, 1953a.

8. *Coscinoptera humeralis* Monrós, 1953a (CAT, CHA, COR, MZA, SAL) Host plant: Fabaceae: *Prosopis* sp. (Roig-Juñent, 2004).

*Euroscopa (Coleoguerina) humeralis*: Moldenke, 1981 (misspelled for *Euryscopa*).

9. *Coscinoptera nigerrima* Guérin, 1945 (COR, SEO).

*Euroscopa (Coleoguerina) nigerrima*: Moldenke, 1981 (misspelled for *Euryscopa*).

10. *Coscinopteraobliqua* Lacordaire, 1848 (CTS).

*obliqua* Lacordaire, 1848 (*incertae sedis* in Moldenke, 1981).

10. *Coscinopteraterebellum* Lacordaire, 1848 (CTS, MNS).

*Euryscopa terebellum*: Monrós, 1953a.

11. *Coscinoptera tibialis* Harold, 1875 (CHA, COR, ERS, FOR, MZA, SAL TUC, SEO). Host plant: Fabaceae: *Acacia caven* (Mol.) Mol. (Monrós, 1953a).

###### Euryscopa

Taxon classificationAnimaliaColeopteraChrysomelidae

Lacordaire, 1848

Euryscopa Lacordaire, 1848: 493–494; Chapuis 1874: 140; Jacoby 1880: 31–32; Horn 1892: 15–16; Jacoby and Clavareau 1906: 64; Clavareau 1913: 78; Guérin 1943: 34–35; Monrós 1953a: 102–103; Moldenke 1970: 74, 1981: 93.

####### Note.

Moldenke (1981) divided this genus into three subgenera, one of these, *E. (Coleoguerina)*, was synonymized by Agrain and Roig-Juñent (2011) with *Coscinoptera*. From the remaining two subgenera only one has representative species in Argentina, E. (Coleomonrosa).

###### Euryscopa (Coleomonrosa)

Taxon classificationAnimaliaColeopteraChrysomelidae

Moldenke, 1981

Fig. 18

####### Type species.

*Euryscopa semicincta* Lacordaire, 1848, designated by Moldenke 1981: 94.

**Figure 18.**
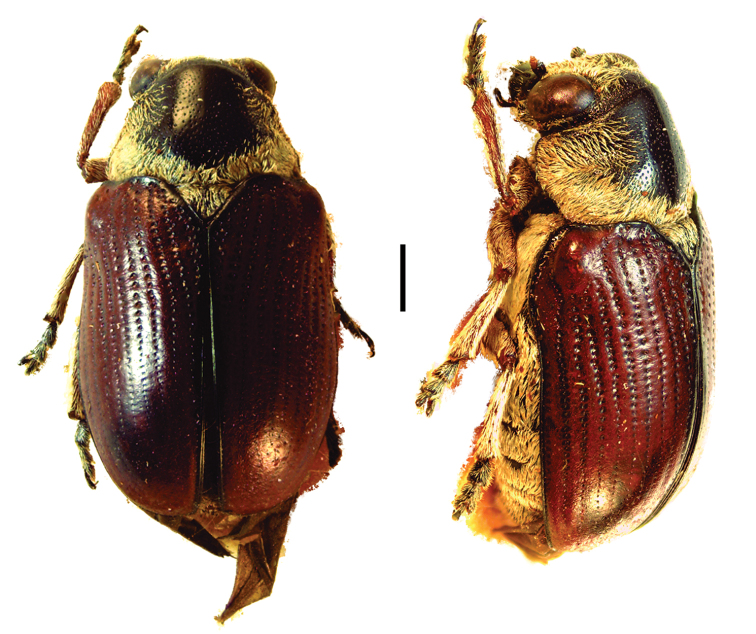
Euryscopa (Coleomonrosa) haematoptera Lacordaire ^(1)^, left: habitus (dorsal view), right: habitus (lateral view).

####### Diagnosis.

Size greater than 7mm; robust, widest at humeral angle; scutellum foveate; aedeagus occupying entire abdominal length, distinctly straight; body not metallic, eyes weakly emarginate; antennomere IV smaller than V; elytral punctation seriate or sub-seriate, without pubescence; pygidium with transverse subapical carina.

####### Distribution.

Argentina, Brazil, Colombia, Costa Rica, French Guiana, Mexico, Peru, Suriname, and Venezuela.

####### Argentinian species checklist.

1. Euryscopa (Coleomonrosa) haematoptera Lacordaire, 1848 (MNS).

*Euryscopa haematoptera* Lacordaire, 1848.

Euryscopa (Coleomonrosa) haematoptera: Moldenke, 1981.

2. Euryscopa (Coleomonrosa) scapularis Lacordaire 1848 (COR, MNS). Host plant: Asteraceae:

*Baccharis* sp. (Monrós 1947).

*Euryscopa scapularis* (Moldenke 1981) Lacordaire, 1848.

Euryscopa (Coleomonrosa) scapularis: Moldenke, 1981.

###### Megalostomis

Taxon classificationAnimaliaColeopteraChrysomelidae

Chevrolat, 1836

Fig. 19

Megalostomis Chevrolat 1836: 416; Lacordaire 1848: 519; Blanchard 1851: 534; Chapuis 1874: 135; Gemminger and Harold 1874: 3294; Jacoby 1876: 809, 1880: 29; Horn 1892: 10; Jacoby and Clavareau 1906: 58; Clavareau 1913: 74; Bruch 1914: 348; Guérin 1943: 9; Monrós 1953a: 61, 1953b: 46; Moldenke 1970: 14, 1981: 99.
Megalostomis (Megalostomis) Chevrolat 1836: 416; Lacordaire 1848: 534; Chapuis 1874: 137; Jacoby and Clavareau 1906: 59; Guérin 1943: 15; Monrós 1953a: 71; Moldenke 1970: 19, 1981: 100.
=Megalostomis (Minturnia) Lacordaire 1848: 520; Chapuis 1874: 136; Jacoby and Clavareau 1906: 60; Guérin 1943: 11; Monrós 1953a: 62; Moldenke 1970: 19, 1981: 100; Agrain and Roig-Juñent 2011: 672, 695 (SYN).
=Megalostomis (Heterostomis) Lacordaire 1848: 554; Chapuis 1874: 138; Jacoby and Clavareau 1906: 60; Guérin 1943: 27; Monrós 1953a: 78; Agrain and Roig-Juñent 2011: 672, 695 (SYN).
=Megalostomis (Scaphigenia) Lacordaire 1848: 547; Chapuis 1874: 137; Jacoby and Clavareau 1906: 60; Clavareau 1913: 75; Achard 1926: 148; Guérin 1943: 24; Monrós 1953a: 88; Seeno and Wilcox 1982: 33; Agrain et al. 2007: 340; Agrain and Roig-Juñent 2011: 672, 695 (SYN).
=Megalostomis (Pygidiocarina)Moldenke 1970: 26, 1981: 83; Agrain and Roig-Juñent 2011: 672, 695 (SYN).
=Megalostomis (Coleobyersa) Moldenke 1981: 101; Agrain and Roig-Juñent 2011: 672, 695 (SYN).
=Megalostomis (Snellingia) Moldenke 1981: 101; Agrain and Roig-Juñent 2011: 672, 695 (SYN).

####### Type species.

*Clythra boopis* (Germar, 1824) [= *Megalostomis grossa* (Forsberg 1821)], subsequent designation by Monrós 1953b: 46.

**Figure 19.**
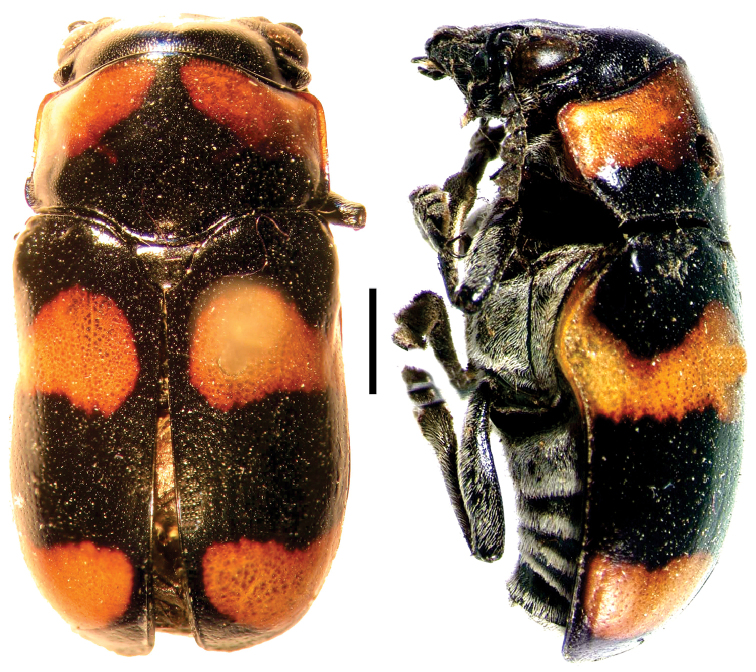
*Megalostomis grossa* (Forsberg) ^(1)^, left: habitus (dorsal view), right: habitus (lateral view).

####### Diagnosis.

Among the species of *Megalostomis*, several morphological differences exist and the head and thorax are highly variable, therefore, the most useful morphological characters are: presence of a carina in the inter-ocular area, development of anterior teeth on the mandibles, clypeus sculpture, and the degree of retraction of the head inside the prothorax. The thorax may have strong constrictions, which are often present in those species showing great development of the head and mouthparts. The elytra are also variable; the most distinctive characters are the coloration pattern and the ordering of the elytral punctation. Although also variable, the abdomen and legs are not especially useful for the recognition of species groups. The pygidium may possess distinct sculpture patterns, which are useful to diagnose among species.

####### Distribution.

*Megalostomis* distribution includes North, Central and South America, especially diverse in xeric temperate or subtropical zones.

####### Remarks.

This genus was revised by Agrain (2013). *Megalostomis* now includes 43 species (Agrain 2014), 13 of them present in Argentina. According to Agrain and Roig-Juñent (2011) this genus is supported by two synapomorphies: eyes strongly emarginated and dorsal plate of aedeagus with straight margin. The morphology in the genus is highly variable, especially for sexual dimorphic features such as: a greater development of mandibles, the presence of constrictions on the pronotal disc, development of double infraocular projection and lengthening of forelegs (Agrain 2013).

####### Argentinian species checklist.

1. *Megalostomis analis* (Forsberg, 1821) (COR, CTS, FOR, MNS, SEO).

= *Clythra analis* Forsberg, 1821.

= *Clythra bicincta* Germar, 1824.

= *Megalostomis bicincta* Germar 1824.

Megalostomis (Heterostomis) analis: Lacordaire, 1848.

= Megalostomis (Heterostomis) analis var seminigra Achard, 1926.

= Megalostomis (Heterostomis) analis var lateralis Achard, 1926.

2. *Megalostomis consimilis* Achard, 1926 (CAT, CHA, COR, CTS, FOR, JUY, LRA, MZA, SAL, SEO, SLS, TUC). Host plants: Fabaceae: *Prosopis algarrobilla* (Roig-Juñent 2004), *Prosopis affinis* (Roig-Juñent 2004), *Prosopis* sp. (Viana and Williner 1974).

Megalostomis (Scaphigenia) consimilis: Agrain et al., 2007.

= Megalostomis (Scaphigenia) cornuta Monrós, 1945 (nec Lacordaire, 1848).

Megalostomis (Scaphigenia) cornuta consimilis: Monrós 1956a.

3. *Megalostomis cornuta* Lacordaire, 1848 (COR, SAL, SEO).

= *Megalostomis cornuta* Dejean, 1836 (*nomen nudum*).

Megalostomis (Scaphigenia) cornuta Lacordaire, 1848.

= Megalostomis (Scaphigenia) cornuta var. baeri Achard, 1926.

= Megalostomis (Scaphigenia) cornuta var. obliterate Achard 1926.

= Megalostomis (Scaphigenia) cornuta var. divisa Guérin, 1949.

4. *Megalostomis gazella* Lacordaire, 1848 (CAT, CHA, COR, CTS, ERS, FOR, JUY, LPA, LRA, MNS, MZA, SAL, SEO, SFE, SJN, SLS, TUC). Host plants: Fabaceae: *Prosopis* sp.; *Acacia* sp. (Monrós 1953a); *Prosopis caldenia* (Aravena 1974), *Prosopis flexuosa* (Roig-Juñent 2004). Ant hosts: Colonies of *Camponotus* sp. (Monrós 1953a, as Dr. Oblobin pers. comm.)

Megalostomis (Scaphigenia) gazella Lacordaire, 1848.

= Megalostomis (Scaphigenia) gazella var. clavapex Achard, 1926.

= Megalostomis (Scaphigenia) gazella var. flavapex: Monrós, 1953a (misspelling pro clavapex).

= Megalostomis (Scaphigenia) gazella var. nigrapex Achard, 1926.

= Megalostomis (Scaphigenia) gazella var. nigrescens Achard, 1926.

= *Megalostomis meretrix* Lacordaire, 1848.

= *Megalostomis bicingulata* Lacordaire, 1848.

5. *Megalostomis grossa* (Forsberg, 1821) (COR, CTS, FOR, JUY, MNS) .

= *Clythra grossa* Forsberg, 1821.

= *Clythra boopis* Germar, 1824.

= *Megalostomis boopis*: Dejean, 1836.

= *Megalostomis interrupta* Dejean, 1836 (*nomen nudum*).

Megalostomis (Megalostomis) grossa Lacordaire, 1848.

= Megalostomis (Megalostomis) grossa brasiliana Achard, 1926.

= Megalostomis (Megalostomis) grossa cinctipennis Achard, 1926.

= Megalostomis grossa var. boopis Achard, 1926.

= Megalostomis grossa var. quadrimaculata Achard, 1926.

6. *Megalostomis kollari* Lacordaire, 1848 (COR, MNS).

Megalostomis (Scaphigenia) kollari Lacordaire, 1848.

7. *Megalostomis lacordairei* Lacordaire, 1848 (CAT, CHA, CTS, FOR, LRA, MNS, MZA, SAL, SEO, SFE, SJN, SLS, TUC). Host plants: Fabaceae (Monrós 1953a) *Cercidium praecox* (Brea); *Prosopis* sp.; *Geoffroea decorticans* (Chañar) (Viana and Williner 1974); *Acacia caven* (Mol.) Mol. (Viana and Williner 1974); *Senna aphylla* (Agrain and Marvaldi 2009); *Prosopis* sp. and Verbenaceae (Cordo and DeLoach 1995): *Aloysia gratissima*. Zygophyllaceae: *Bulnesia retama* (Common name in Argentina: Retamo, in Peru: Calato).

= *Megalostomis lacordairei* Dejean, 1836 (*nomen nudum*).

Megalostomis (Heterostomis) lacordairei Lacordaire, 1848.

= Megalostomis (Heterostomis) histrionica Harold, 1875.

= Megalostomis (Heterostomis) lacordairei var. seminigra Achard, 1926.

= Megalostomis lacordairei var. basalis Achard, 1926.

= Megalostomis lacordairei var. collaris Achard, 1926.

= Megalostomis lacordairei var. conjuncta Achard, 1926.

= Megalostomis lacordairei var. consimilis Achard, 1926.

= Megalostomis lacordairei var. histrionica Achard, 1926.

= Megalostomis lacordairei var. interrrupta Achard, 1926.

= Megalostomis lacordairei var. reducta Achard, 1926.

8. *Megalostomis querula* Lacordaire, 1848 (CTS, ERS) Host plants: Salicaceae: *Caesaria sylvestris* (Guacatonga or wild coffee) (Agrain 2013).

Megalostomis (Minturnia) querula Lacordaire, 1848.

= Megalostomis (Minturnia) propinqua Lacordaire, 1848.

= Megalostomis (Minturnia) univittata pacifica Monrós, 1953a.

9. *Megalostomis religiosa* Lacordaire, 1848 (CTS, MNS).

= *Megalostomis religiosa* Dejean, 1836 (*nomen nudum*).

Megalostomis (Scaphigenia) religiosa Lacordaire, 1848.

= *Megalostomis distincta* Lacordaire, 1848.

10. *Megalostomis robustipes* Monrós, 1953a (MNS).

=Megalostomis (Minturnia) robustipes Monrós, 1953a.

11. *Megalostomis tricincta* (Germar, 1824) (CTS, MNS).

= *Clythra tricincta* Germar, 1824.

Megalostomis (Megalostomis) tricincta: Lacordaire, 1848.

= Megalostomis (Scaphigenia) bubalus Lacordaire, 1848.

= Megalostomis (Scaphigenia) religiosa Monrós, 1945 (nec Lacordaire, 1848).

= Megalostomis (Scaphigenia) bubalus bubaloides Monrós, 1953a.

12. *Megalostomis univittata* Lacordaire, 1848 (MNS, SAL).

= Megalostomis (Minturnia) univittata univittata Lacordaire, 1848.

= Megalostomis (Minturnia) univittata oblita Monrós, 1953a.

13. *Megalostomis vianai* Monrós, 1947 (MNS).

Megalostomis (Minturnia) vianai Monrós, 1947.

###### Themesia

Taxon classificationAnimaliaColeopteraChrysomelidae

Lacordaire, 1848

Fig. 20

Themesia Lacordaire 1848: 517-518; Chapuis 1874: 138; Jacoby and Clavareau 1906: 62; Clavareau 1913: 77; Guérin 1943: 30; Monrós 1953a: 130; Moldenke 1970: 12.

####### Type species.

*Themesia auricapilla* (Germar, 1824), designated by Monrós 1953a: 130.

**Figure 20.**
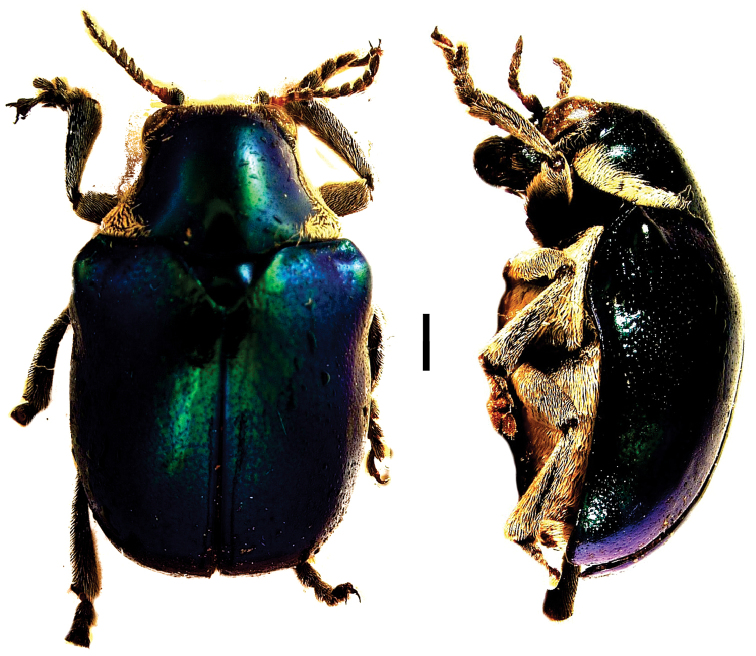
*Themesia auricapilla grandis* Baly ^(1)^, left: habitus (dorsal view), right: habitus (lateral view).

####### Diagnosis.

This genus is close to *Coscinoptera*, from which it can be separated by its unicolored metallic green/blue coloration (except in *T. costaricensis* and *T. lacordairei*, not present in Argentina) and by its distinct body shape. Eyes large, non-emarginate and distinctly protruding; elytra glabrous without distinct punctation pattern, epipleurae reduced; pygidium flat; antennomere IV much smaller than V, not clavate; ventrites densely pubescent.

####### Distribution.

From Costa Rica to Colombia, and in Brazil, Paraguay, and Argentina.

####### Argentinian species checklist.

1. *Themesia auricapilla grandis* Baly, 1877a (CTS, MNS, TUC).

*Themesia grandis* Baly, 1877a.

#### Tribe Cryptocephalini Gyllenhal, 1813

The most common characters to differentiate this tribe are the procoxae globose, distinctly separated by intercoxal prosternal process. In males of many species of *Cryptocephalus* (Cryptocephalina) and *Griburius* (Pachybrachina) the dorsal lobes of the eyes are strongly converging towards the median line and may come into contact with each other. Phylogenetic significance (if any) of this trait is unclear. The antennae are long and filiform in most genera, often reaching the humeral callus or further, although there are exceptions (eg. clavate in Fulcidacini).

#### Subtribe Cryptocephalina Gyllenhal, 1813

The most distinctive character is the crenulate, not margined, base of pronotum. Some characters present variation, such as the tarsal claws which may be simple or appendiculate, or antennae, which may be short and clavate to subserrate.

##### Cryptocephalus

Taxon classificationAnimaliaColeopteraChrysomelidae

Geoffroy, 1762

Fig. 21

Cryptocephalus Geoffroy, 1762: 231 (conserved name); Chevrolat 1836: 422; Saunders 1845: 142; Redtenbacher 1845: 118; Gistel 1848: 123; Haldeman 1849: 170; Stål 1857: 61; Chapuis 1874: 184; Jacoby 1880; 42; Baly 1877b: 32; Burmeister 1877: 64; Burlini 1953: 75; Lopatin 1965: 452; White 1968: 24; Blackwelder 1946: 644.
Cryptocephalus Müller, 1764 (subsequent use).
=Physicerus Chevrolat, 1836.
=Strigogophorus Chevrolat, 1836 (nomen nudum).
=Dicenopsis Saunders, 1842.
=Mitocera Saunders, 1842.
=Ochrosopsis Saunders, 1843.
=Anodonta Saunders, 1845, not Lamarck 1799 (Mollusca).
=Idiocephala Saunders, 1845.
=Ochrosopsus : Saunders, 1845 (error).
=Canthostethus Haldeman, 1849.
=Mecostethus Stål, 1857.
=Euphyma Baly, 1877c.

###### Type species.

*Chrysomela sericea*, designated by Latreille 1810: 432.

**Figure 21.**
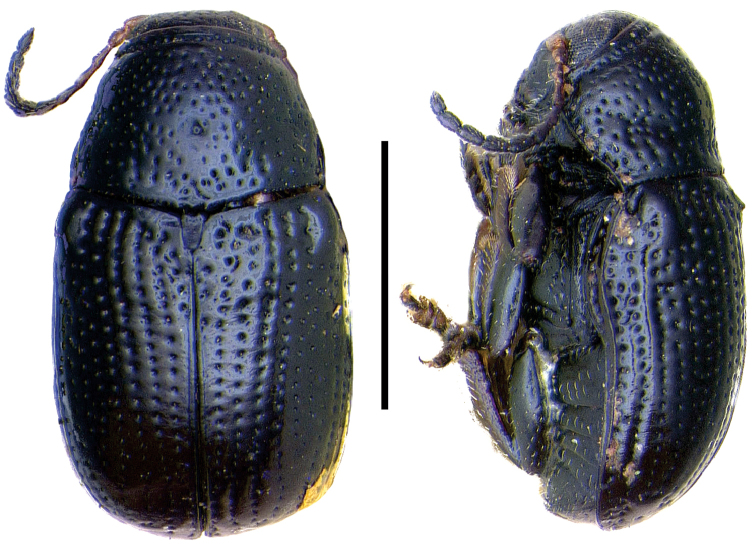
*Cryptocephalus carbonarius* Burmeister ^(2)^, left: habitus (dorsal view), right: habitus (lateral view).

###### Diagnosis.

Anteriorly flat head, deeply inserted into the prothorax; eyes reniform; leading edge of prothorax laterally straight; denticles present on posterior margin of pronotum; thorax closely fitted to base of elytra (thus sometimes concealing denticles); anterior margin of intercoxal prosternal process uniformly concave or with medial flange; intercoxal width equal to or greater than width of coxal cavity; ventrite I of male without spines. Rectal apparatus bearing one ventral and two dorsal sclerites.

###### Distribution.

Worldwide, with over 1700 species (Chamorro 2014b), with nine species cited for Argentina.

###### Remarks.

Although a complex subgeneric classification does exist for Palearctic species (Schöller 2002), new world species including Argentinian, have not yet been assigned to subgenera.

###### Argentinian species checklist.

1. *Cryptocephalus acuminatus* Jacoby, 1907 (TUC).

2. *Cryptocephalus argentinus* Jacoby, 1907 (BAS).

3. *Cryptocephalus carbonarius* Burmeister, 1877 (BAS).

4. *Cryptocephalus fusculus* Suffrian, 1863 (BAS).

5. *Cryptocephalus incommodus* Suffrian, 1863 (BAS).

6. *Cryptocephalus misellus* Suffrian, 1857 (BAS, CTS).

7. *Cryptocephalus tucumanensis* Jacoby, 1907 (TUC).

8. *Cryptocephalus subaenescens* Jacoby, 1907 (TUC).

#### Subtribe Monachulina Leng, 1920

The members of this subtribe have the intercoxal prosternal process noticeably wider than long; tarsal claws appendiculate; antennae are usually short (rarely longer than base of pronotum) and antennomeres expanded laterally.

##### Lexiphanes

Taxon classificationAnimaliaColeopteraChrysomelidae

Gistel, 1848

Fig. 22

Lexiphanes Gistel, 1848: 123; Balsbaugh 1966: 660.
=Monachus Chevrolat 1836 (not Kaup 1829, not Flemming 1822).
=Monachulus Leng 1918.

###### Type species.

*Cryptocephalus saponatus* Fabricius [= *Lexiphanes saponatus* (Fabricius)], designated by Balsbaugh (1966: 660).

**Figure 22.**
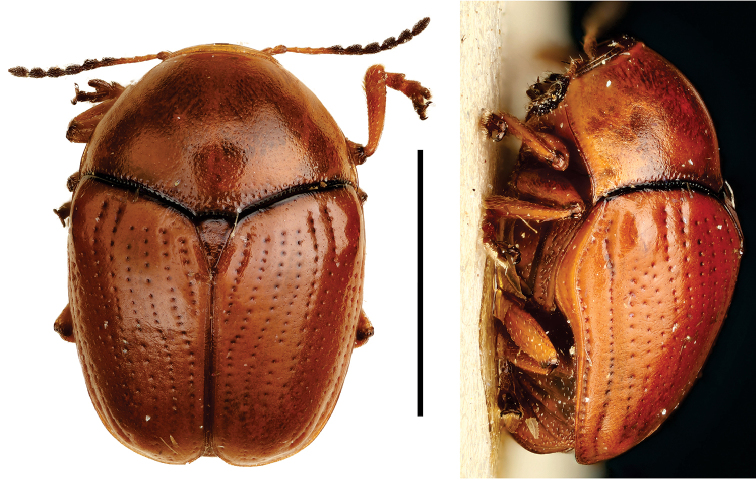
*Lexiphanes consimilis* (Suffrian) ^(2)^, left: habitus (dorsal view), right: habitus (lateral view).

###### Diagnosis.

*Lexiphanes* may be most commonly confused with *Stegnocephala* and less so with *Cryptocephalus*. Both genera in Monachulina have shorter antennae (rarely surpassing half of entire body length) with antennomeres anteriorly expanded (less so in *Lexiphanes*). Also, the intercoxal prosternal process is wide and bilobed with small lateral projections and the anterior margin of intercoxal prosternal process uniformly concave (Chamorro-Lacayo and Konstantinov 2004). *Lexiphanes* can be distinguished from *Stegnocephala* by the more uniform rounded shape of the pronotum, which lacks basolateral depressions. The prothoracic anterior opening in *Lexiphanes* has a circumference, best viewed anteriorly, with the dorsal and ventral margins on the similar vertical plane (in lateral view). In general, *Stegnocephala* is larger, more robust, and colorful than *Lexiphanes*.

###### Distribution.

This genus is restricted to the New World, from México to Argentina with over 100 species. 11 of which are present in Argentina.

###### Remarks.

Balsbaugh (1966) revised the North American species of this genus. The limits of the subtribe, genera, and species need revision. Information is lacking for Central and South American species that are known only from their original descriptions in the 19^th^ century. The presence of denticles on the posterior margin of pronotum is shared with Cryptocephalina, therefore Monachulina may not be a natural group, and may be a synonym of Cryptocephalina. This hypothesis remains to be tested.

###### Argentinian species checklist.

1. *Lexiphanes anthracinus* (Burmeister, 1877) (Patagonia, RNO).

2. *Lexiphanes biplagiatus* (Boheman, 1858) (BAS, CTS).

3. *Lexiphanes coenobita* (Suffrian, 1863) (TUC).

4. *Lexiphanes consimilis* (Suffrian, 1863) (BAS).

5. *Lexiphanes ebeninus* (Burmeister, 1877) (SCZ).

6. *Lexiphanes flavifrons* (Burmeister, 1877) (Patagonia, SCZ).

7. *Lexiphanes granarius* (Suffrian, 1863) (Argentina).

8. *Lexiphanes modestus* (Boheman, 1858) (Argentina).

9. *Lexiphanes nigritulus* (Boheman, 1858) (BAS).

10. *Lexiphanes ornatipennis* (Jacoby, 1908) (TUC).

11. *Lexiphanes saucius* (Burmeister, 1877) (BAS).

##### Stegnocephala

Taxon classificationAnimaliaColeopteraChrysomelidae

Baly, 1877

Fig. 23

Stegnocephala Baly 1877b: 32; Jacoby 1889: 122; Clavareau 1913: 113; Bruch 1914: 352.

###### Type species.

*Cryptocephalus hemixanthus* Suffrian, by original designation.

**Figure 23.**
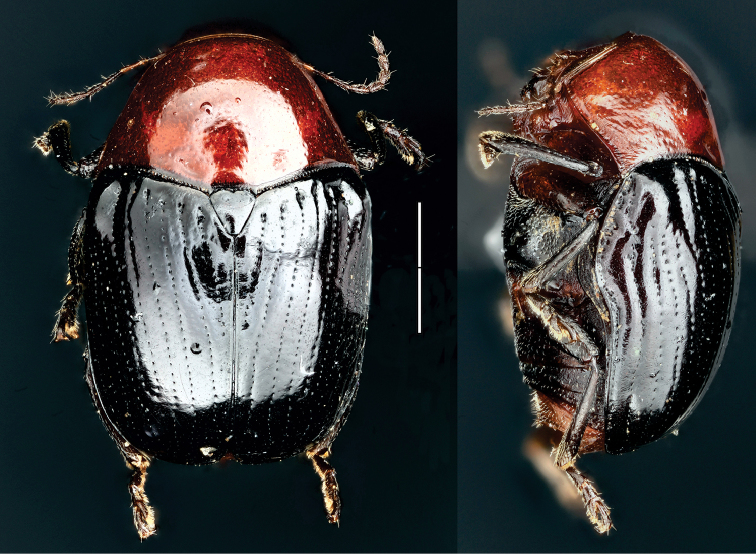
*Stegnocephala xanthopyga* (Suffrian) ^(2)^, left: habitus (dorsal view), right: habitus (lateral view).

###### Diagnosis.

Coxa widely separated, epipleural lobes strongly produced. Chamorro-Lacayo and Konstantinov (2004), provided several prothoracic characters: pronotal punctures absent; intercoxal prosternal process truncate; anterior margin of intercoxal prosternal process with a medial flange, or two submedial flanges.

###### Distribution.

From Costa Rica to Argentina, mainly in tropical regions.

###### Remarks.

Weise (1921) disagreed with the separation of this genus from *Cryptocephalus*. White (1968), interpreted Weise’s comment as the synonymyzation of *Stegnocephala* with *Cryptocephalus*. Since Weise (1921) only provided morphological differences of *Cryptocephalus perplexus* Suffrian, which is not the type species of the genus, we still consider *Stegnocephala* as a valid genus. Chamorro is currently revising the genus.

###### Argentinian species checklist.

*Stegnocephala discoidalis* Baly, 1877c (MNS).

*Stegnocephala xanthopyga* (Suffrian, 1863) (TUC). This species newly cited for Argentina (Tucuman, Famaillá: Quebrada de Lules, 30-I-1942 // F. Monrós Collection 1959).

#### Subtribe Pachybrachina Chapuis, 1874

The following characters (when combined) can help with the identification of its members (Chamorro 2013): Presence of tibial spurs (absent in *Mylassa*, *Ambrotodes*, and *Griburius* *s. str.*); lack of denticles on the posterior margin of the pronotum (i.e. not crenulate); base of pronotum margined and bilobed sinuate (except in *Mylassa* and less or differently margined in *Ambrotodes*); coarsely punctate dorsally and ventrally including hypomeron (except *Sternoglossus*, and *Mylassa*); confused elytral punctures (except *Mylassa*; less orderly in *Griburius*, *Metallactus*); intercoxal prosternal process lobed (bilobed in other groups) and posterior margin produced caudad (less so in *Pachybrachis*; eyes visible from above (not visible from above in *Mylassa* and *Ambrotodes*; bulging, particularly in *Ambrotodes*, and *Mylassa*, in all other genera the dorsal section of the eye is generally larger than the ventral part as separated by the well developed canthus (canthus weak in *Ambrotodes*, and *Mylassa*. This subtribe is currently being revised by Davide Sassi.

##### Griburius

Taxon classificationAnimaliaColeopteraChrysomelidae

Haldeman, 1849

Fig. 24

Griburius Haldeman, 1849: 245.
=Scolochrus Suffrian, 1852.

###### Type species.

*Griburius scutellaris* Haldeman 1849: 245 (= *Cryptocephalus scutellaris* Fabricius, 1801), by monotypy.

**Figure 24.**
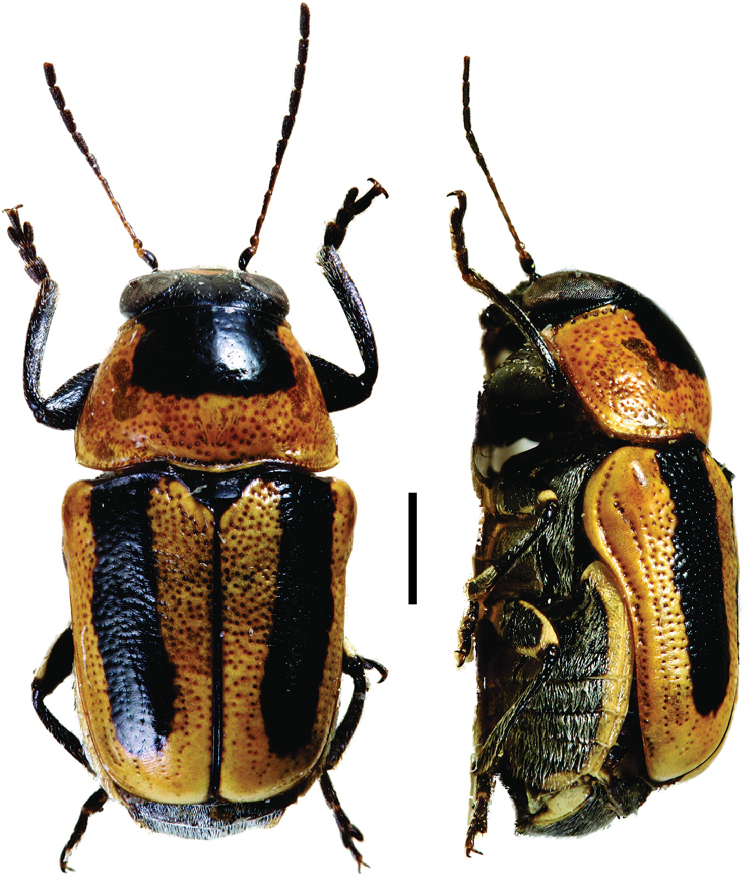
*Griburius cultus* (Suffrian) ^(4)^, left: habitus (dorsal view), right: habitus (lateral view).

###### Diagnosis.

Recently, Chamorro (2013) provided the following characters to identify the species of this genus: posterior margin of intercoxal prosternal process rounded; lateral margin of elytra deeply excised exposing abdomen caudally, elytra length approximately 2× or less length of pronotum. However, the limits of *Griburius* and *Metallactus* are confused and require revision.

###### Distribution.

Nearctic and Neotropical.

###### Argentinian species checklist.

*Griburius bilineolatus* (Suffrian, 1866) (BAS, COR).

*Griburius conspurcatus* (Suffrian, 1866) (BAS).

*Griburius cultus* (Suffrian, 1866) (BAS).

*Griburius fastidiosus* (Suffrian, 1866) (BAS).

*Griburius octoguttatus* Burmeister, 1877 (ERS).

*Griburius persimilis* Burmeister, 1877 (BAS).

##### Metallactus

Taxon classificationAnimaliaColeopteraChrysomelidae

Suffrian, 1866

Fig. 25

Metallactus Suffrian 1866: 248; Jacoby 1907: 848.

###### Type species.

Not yet designated.

**Figure 25.**
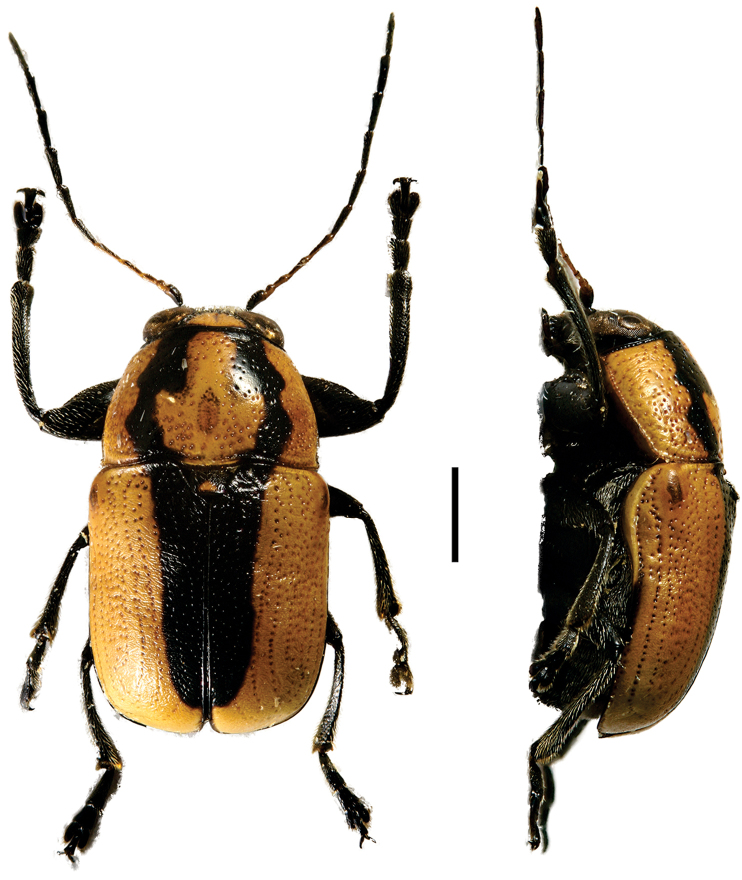
*Metallactus argentinensis* Jacoby ^(4)^, left: habitus (dorsal view), right: habitus (lateral view).

###### Diagnosis.

*Metallactus* includes species that lack a deeply excised lateral edge of the elytra, additionally, the following characters may be useful to segregate species into this genus: posterior margin of intercoxal prosternal process gradually narrowing, pointed; abdomen not exposed; elytral length greater than 2× length of pronotum (Chamorro 2013).

###### Distribution.

Neotropical.

###### Remarks.

This genus has not been revised since its original description by Suffrian (1866) and its relationship with related genera is presently unclear (Schöller 2003, Sassi 2015). Furthermore, Jacoby (1907) indicated *Metallactus* and *Griburius* to be very problematic to define, and a lot of species can not fit well in either genera. However, a study with a new diagnosis of the genus *Metallactus*, based on a new set of effective morphological characters is in progress (Sassi, *in prep.*).

###### Argentinian species checklist.

1. *Metallactus albipes* Suffrian, 1866 (CTS).

2. *Metallactus albopictus* Suffrian, 1866 (BAS, COR).

3. *Metallactus argentinensis* Jacoby, 1907 (COR).

4. *Metallactus bivitticollis* (Jacoby, 1907), **comb. n.** (BAS).

5. *Metallactus divisus* Jacoby, 1907 (SFE).

6. *Metallactus generosus* Suffrian, 1866 (CTS).

7. *Metallactus inustus* Suffrian, 1866 (BAS, CTS).

8. *Metallactus luniger* Suffrian, 1866 (Argentina).

9. *Metallactus nigrofasciatus* Suffrian, 1866 (COR, SLS). Host plant: Asteraceae: carqueja (*Baccharis* sp.) (Viana and Williner 1973).

10. *Metallactus nigrovittis* Jacoby, 1907 (SFE).

11. *Metallactus patagonicus* Suffrian, 1866 (BAS, COR, MZA, RNO).

12. *Metallactus pollens* Suffrian, 1866 (CTS).

##### Mylassa

Taxon classificationAnimaliaColeopteraChrysomelidae

Stål, 1857

Fig. 26

Mylassa : Stål 1857: 60; Baly 1877b: 32; Jakobson 1924: 258 (placed in Pachybrachina); Monrós 1949a: 492 (placed in Cryptocephalina); Schöller and Heinig 2003: 9, = Cryptocephalus; Suffrian 1863: 174; Blackwelder 1946: 644; Jerez and Briones 2010: 32.

###### Type species.

*Mylassa fasciatipennis* Stål (=*Pachybrachis crassicollis* Blanchard), designated by Jakobson 1924: 258.

**Figure 26.**
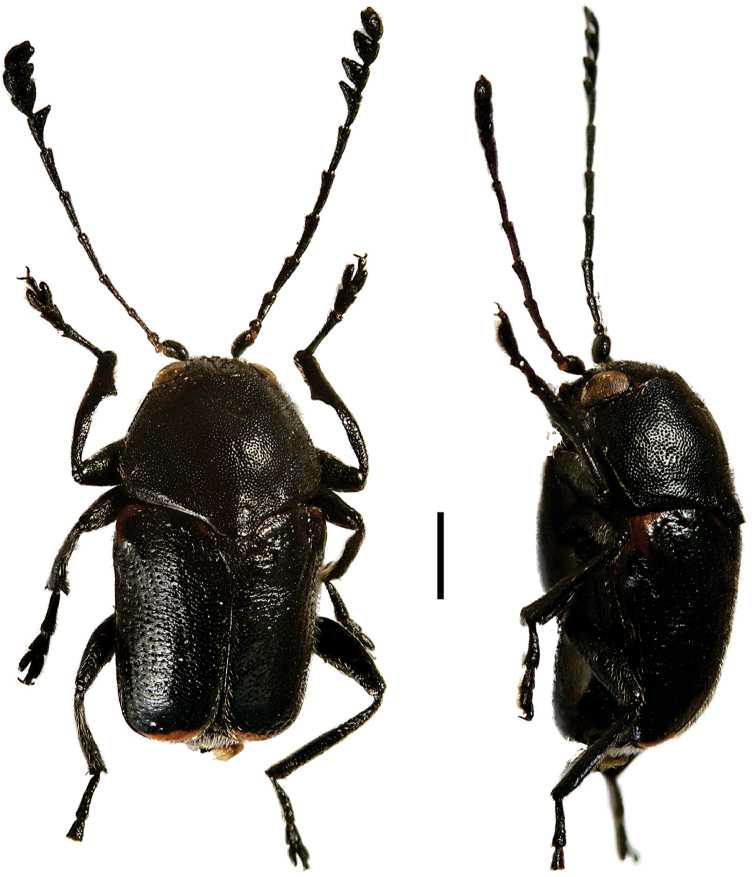
*Mylassa pectinicornis* (Suffrian) ^(4)^, left: habitus (dorsal view), right: habitus (lateral view).

###### Diagnosis.

This genus can be easily distinguished from all others in the area by the presence of pubescence on its body and by the presence of a basal thoracic lobe with raised, thickened apex. Furthermore, it has nearly entire eyes and the rectal apparatus bears two ventral and three dorsal sclerites, with the shape of the dorsal central plate band-like (very narrow) (Reid 1990; Schöller 2008).

###### Distribution.

This genus has eight species described form Southern Chile and Argentina, and some new species awaiting description. Species are found between 30°S and 42°S and are associated with sclerophyllous shrubs (Jerez and Briones 2010).

###### Remarks.

This genus was considered a synonym of *Cryptocephalus* by several authors, however several studies support its validity and it is hypothesized to be included in Pachybrachina (Baly 1877b; Jakobson 1924; Monrós 1949a, Schöller 2008; Jerez and Briones 2010) or in its own subtribe (Reid 1990; Chamorro and Konstantinov, unpublished data).

###### Argentinian species checklist.

1. *Mylassa chachallaoi* Monrós, 1949a (CHU, RNO). Host plant: Proteaceae: *Lomatia obliqua* (Monrós 1949a).

2. *Mylassa crassicollis* (Blanchard, 1851) (NQN, RNO). Host plant: Anacardiaceae: “litrenillo”, *Schinus*? (Bosq 1943), *Schinus* sp. (Monrós 1949a); Betulaceae: *Betula* sp., Elaeocarpaceae: *Aristotelia* sp., and *Aristotelia maqui*, *Aristotelia chilensis* (as main food source) (Monrós 1949a), Ericaceae: *Gaultheria* sp. (Monrós 1949a), *Pernettya* sp. (Bosq 1943), Salicaceae: *Populus* sp. (Monrós 1949a).

= *Mylassa fasciatipennis* Stål, 1857.

3. *Mylassa discariana* Monrós, 1949a (RNO). Host plant: Rhamnaceae: *Discaria* sp. (Monrós 1949a).

4. *Mylassa frigens* Monrós, 1949a (NQN).

5. *Mylassa obliquata* (Suffrian, 1863) (NQN, RNO).

6. *Mylassa pectinicornis* (Suffrian, 1866) (NQN, RNO, CHU). Host plant: Proteaceae: *Lomatia obliqua* (Monrós 1949a).

##### Pachybrachis

Taxon classificationAnimaliaColeopteraChrysomelidae

Chevrolat, 1836

Fig. 27

Pachybrachis : Chevrolat, 1836: 420.
=Pachybrachis Redtenbacher, 1845.
=Pachystylus Rey, 1883.
Pachybrachys : Mannerheim 1843: 311. Incorrect subsequent spelling.

###### Type species.

*Cryptocephalus hieroglyphicus* Laicharting, 1781. By subsequent designation of Jacoby 1908: 265.

**Figure 27.**
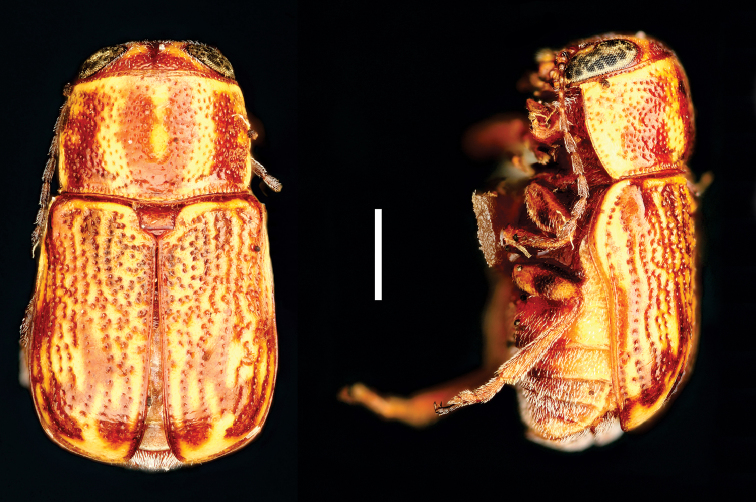
*Pachybrachis mysticus* Suffrian ^(2)^, left: habitus (dorsal view), right: habitus (lateral view).

###### Diagnosis.

Posterior margin of intercoxal prosternal process relatively entire, rarely produced beyond posterior margin of prothorax; gestalt cylindrical (height of each elytron approximately 2.5 width), pronotum narrower than elytral bases combined, overall flattened not vaulted; punctation on head, prothorax and elytra evident, large; elytral punctation commonly confused (but punctation in rows not uncommon); fore-femora may or may not be enlarged; mesotibiae usually with terminal spur in both sexes.

###### Distribution.

Neartic, Neotropical, Palearctic, and Oriental regions.

###### Remarks.

A subgeneric classification exists for Palearctic species, Neotropical species have not yet been assigned to subgenera.

###### Argentinian species checklist.

1. *Pachybrachis foetidus* Suffrian, 1866 (BAS)

2. *Pachybrachis gayi* Blanchard, 1851 (ARGENTINA). Host plant: Fagaceae: *Nothofagus* and *Castanea* (Jolivet, 1978).

3. *Pachybrachis mysticus* Suffrian 1866 (BAS, LPA). Host plant: Fabaceae: *Prospis caldenia* (Aravena 1974).

4. *Pachybrachis nigronotatus* Boheman, 1858 (BAS)

5. *Pachybrachis xanthogrammus* Suffrian, 1866 (CTS, ERS).

#### Tribe Fulcidacini Jakobson, 1924

= Chlamisini Gressitt, 1946

This tribe is the least diverse within Cryptocephalinae, and it is much more diverse in the Neotropics (Chamorro 2014b). Most adults resemble caterpillar droppings. Chamorro-Lacayo and Konstantinov (2009), undertook a comprehensive synoptic study of the world genera of Fulcidacini.

##### Aulacochlamys

Taxon classificationAnimaliaColeopteraChrysomelidae

Monrós, 1951c

Fig. 28

Aulacochlamys Monrós, 1951c: 657; Chamorro-Lacayo and Konstantinov 2009: 66.

###### Type species.

*Exema costicollis* Lacordaire, 1848, by original designation.

**Figure 28.**
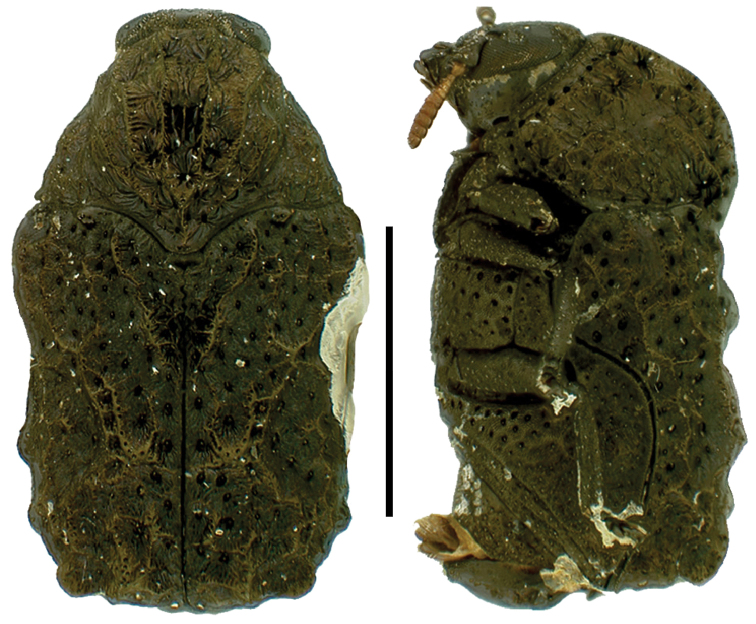
*Aulacochlamys costicollis* (Lacordaire) ^(2)^, left: habitus (dorsal view), right: habitus (lateral view).

###### Diagnosis.

The most salient feature of this genus is the presence medially on the pronotum of six elevated distinct, small, sharp, longitudinal carinae, which converge medially near the posterior margin, reminiscent of a fan. These are small beetles (less than 3 mm length), cylindrical; with antennae serrated beyond antennomere V. antennomeres III-V slightly widened, but not dilated distally; pronotal base opposite mesoscutellum (posterior pronotal lobe) with or without notch; intercoxal prosternal process gradually narrowing posteriorly, broadening before apex; metascutellum concealed by elytra; elytral suture completely serrate, although serration may be weak near scutellum, elytral tubercles well developed. Tibiae slightly curved, cylindrical. *Aulacochlamys* can easily be distinguished from *Chlamisus* Rafinesque by the presence of the six longitudinal carinae on its pronotum.

###### Distribution.

Pantropical, except Australia (Monrós 1951c). Six of the 21 Neotropical species are present in Argentina.

###### Argentinian species checklist.

1. *Aulacochlamys costicollis* (Lacordaire, 1848) (CTS, JUY, MNS).

2. *Aulacochlamys minuta* Monrós, 1951c (MNS).

3. *Aulacochlamys pygidialis* Monrós, 1951c (MNS).

4. *Aulacochlamys radiata* Monrós, 1951c (MNS).

5. *Aulacochlamys rectecarinata* Monrós, 1951c (CTS, MNS, TUC).

6. *Aulacochlamys ultima* Monrós, 1951c (COR).

##### Chlamisus

Taxon classificationAnimaliaColeopteraChrysomelidae

Rafinesque, 1815

Fig. 29

Chlamisus Rafinesque, 1815: 116; Chamorro-Lacayo and Konstantinov 2009: 71.
=Chlamys Knoch, 1801: 122.
=Myochlamys Ihering, 1907.
=Arthrochlamys Ihering, 1904.
=Boloschesis Jakobson, 1924.

###### Type species.

*Chlamys foveolata* Knoch, 1801, by subsequent designation of Navajas 1946: 244 [preoccupied by *Chlamys* Röding, 1798, Mollusca].

**Figure 29.**
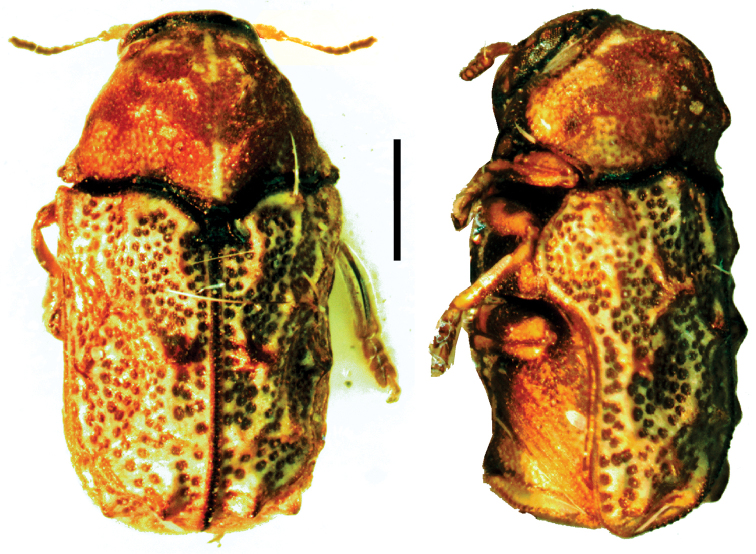
*Chlamisus apricarius* (Lacordaire) ^(3)^, left: habitus (dorsal view), right: habitus (lateral view).

###### Diagnosis.

This genus can be separated from *Exema* Lacordaire by the following characters: males without spines or spinulae on ventrite I; antennomere V nearly as large as VI; sutural serration of elytra usually incomplete (suture entire immediately following mesoscutellum); intercoxal prosternal process posteriorly pointed (narrowed), posteriorly much narrower than anterior margin (Chamorro-Lacayo and Konstantinov 2009). Intermediate size (3-8 mm length). Body usually not metallic in color; elytra without velvety spots. Antenna serrate beyond antennomeres III or IV, antennomere II slightly widened, globose, antennomere V nearly as large as 6^th^. Pronotum medially elevated, with various bumps and short carinae; posterior pronotal lobe with well-differentiated notch; metascutellum not exposed.

###### Distribution.

Cosmopolitan, with over 400 species described worldwide (Monrós 1951c; Reid 1991).

###### Argentinian species checklist.

1. *Chlamisus achalay* Monrós, 1951c (LRA, SAL).

2. *Chlamisus aeronauticus* Monrós, 1951c (JUY, SAL, TUC).

3. *Chlamisus apricarius* (Lacordaire, 1845) (CHT, NQN, RNO).

= *Chlamys fulvescens* Blanchard, 1851.

= *Chlamys minuta* Philippi & Philippi, 1864.

= *Chlamys picta* Philippi & Philippi, 1864.

4. *Chlamisus clarapex* Monrós, 1951c (MNS).

5. *Chlamisus coya* Monrós, 1951c (JUY).

6. *Chlamisus discalceatus* Monrós, 1951c (CHA).

7. *Chlamisus discipennis* (Jacoby, 1901) (MNS). Host plant: Sterculiaceae: *Waltheria americana* (Bokermann 1963).

8. *Chlamisus echinatus* (Klug, 1824) (SAL) Host plant: Euphorbiaceae: *Croton pohlianus* (Bokermann 1963).

9. *Chlamisus gibbicollis* (Lacordaire, 1848) (BAS, CHA, COR, ERS, FOR, JUY, MNS, SAL, TUC)

= *Chlamys lebasii* Lacordaire, 1848. Host plant: Sterculiaceae: *Waltheria americana* (Bokermann 1963).

10. *Chlamisus guarani* Monrós, 1951c (CTS).

11. *Chlamisus hirtus* (Kollar, 1824) (CTS, MNS). Host plants: Fabaceae, Sapindaceae, Malvaceae, Sterculiaceae, Euphorbiaceae, (Monrós 1951c).

12a. *Chlamisus hispidulus hispidulus* (Klug, 1824) (BAS, CHA, COR, FOR, JUY, LRA, MNS, SAL, SEO, SFE, SLS, TUC).

= *Chlamys cordovensis* Jacoby, 1901. Host plants: Fabaceae: *Acacia* sp., *Acacia cavenia*; Asclepiadaceae: “Tasi” (Monrós 1951c).

12b. *Chlamisus hispidulus llajtamaucanus* Monrós, 1951c (COR, LRA, MZA, SEO).

13. *Chlamisus impressus* (Fabricius, 1801) (MNS).

14. *Chlamisus inopinatus* Monrós, 1951c (CTS).

15. *Chlamisus integrithorax* Monrós, 1951c (MNS).

16. *Chlamisus kammerlacheri* (Kollar, 1824) (MNS).

17. *Chlamisus kurkuncho* Monrós, 1951c (JUY, SAL).

18. *Chlamisus langsdorfii* (Kollar, 1824) (MNS).

= *Chlamys rugosa* Klug, 1824. Host plant: Fabaceae: *Bauhinia rufa* (Bokermann, 1963).

19. *Chlamisus longicornis* Monrós, 1951c (MNS).

20. *Chlamisus melochiae* Monrós, 1951c (COR, ERS, SAL, TUC). Host plant: Malvaceae: *Sphaeralcea* sp., Sterculiaceae: *Melochia* sp. (Monrós, 1951c), *Waltheria americana* (Bokermann 1963).

21. *Chlamisus mimicus* Monrós, 1950 (BAS, COR, CTS). Host plant: Melastomaceae: *Tibouchina* sp. (Bokermann 1963).

22. *Chlamisus olivaceus* (Kollar, 1824) (FOR).

= *Chlamys bicolor* Klug, 1824.

23. *Chlamisus pilaga* Monrós, 1951c (FOR). Host plant: Sapindaceae: *Serjaria* sp. (Monrós 1951c).

24. *Chlamisus perforatus* Monrós, 1951c (MNS).

25. *Chlamisus pilicollis* Monrós, 1951c (MNS).

26. *Chlamisus proseni* Monrós, 1951c (JUY).

27a. *Chlamisus puncticollis* (Germar, 1824) (JUY).

= *Chlamys muhlfeldii* Kollar, 1824. Host plant: Sapindaceae: *Serjaria* sp. (Monrós, 1951c).

27b. *Chlamisus puncticollis indigaceus* (Lacordaire, 1848) (COR, MNS).

28. *Chlamisus scortillus* (Lacordaire, 1848) (CTS).

= *Chlamys scortillum* Lacordaire 1848. Host plant: Malpighiaceae: *Banisteria laevigata*, *Banisteria campestris* and *Banisteria crotonifolia* (Bokermann 1963).

29. *Chlamisus scrobicollis* (Lacordaire, 1848) (MNS, SAL).

30. *Chlamisus sidae* Monrós, 1951c (CHA, COR, CTS, FOR, JUY, MNS, SAL, TUC). Host plant: Malvaceae: *Sida rhombifolia* (Monrós, 1951c).

31. *Chlamisus sordidulus* Monrós, 1951c (CHA, CTS, FOR, JUY, MNS, SAL, SFE, TUC).

32. *Chlamisus sulcatus* (Kollar, 1824) (MNS).

= *Chlamys cinnamomea* Klug, 1824. Host plant: Malpighiaceae: *Heteropteris seringiifolia* (Bokermann 1963).

33. *Chlamisus tucumanus* Monrós, 1951c (JUY, SAL, TUC). Host plant: Euphorbiaceae: *Croton* sp. (Monrós 1951c).

34. *Chlamisus vianai* Monrós, 1951c (MNS).

##### Exema

Taxon classificationAnimaliaColeopteraChrysomelidae

Lacordaire, 1848

Fig. 30

Exema Lacordaire, 1848: 844; Jacoby 1908: 278; Karren 1966: 647; Chamorro-Lacayo and Konstantinov 2009: 74.

###### Type species.

*Chlamys intricata* Kollar, 1824, by subsequent designation.

**Figure 30.**
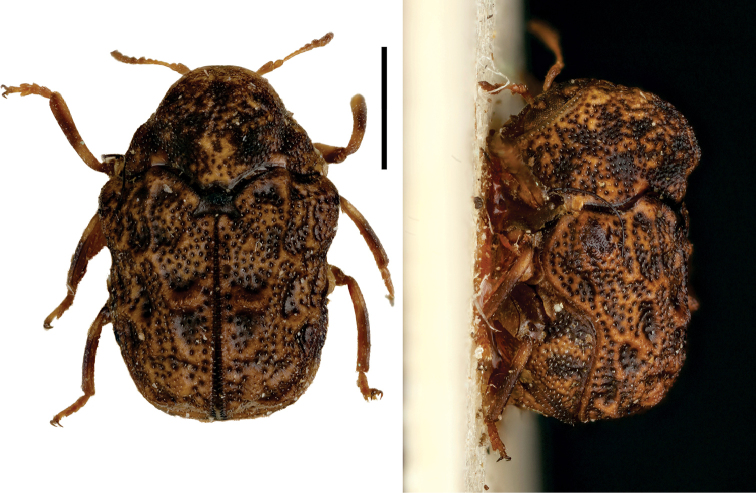
*Exema variopicta* Monrós ^(2)^, left: habitus (dorsal view), right: habitus (lateral view).

###### Diagnosis.

Small species (2–3.5 mm length), cylindrical with widest near shoulders; antenna serrate beyond antennomere V, antennomeres III-IV slightly widened, but not dilated distally; pronotum with various bumps and short ridges, posterior pronotal lobe concave, usually without well differentiated notch.

###### Distribution.

Present in Nearctic, Neotropical, and Oriental regions (Monrós 1951c; Karren 1966). Includes 26 species, 10 represented in the Neotropics.

###### Remarks.

Gressitt and Kimoto (1961) synonymized this genus with *Chlamisus*, yet, this decision has been ignored and is considered to be a valid genus (Karren 1966, 1972; Seeno and Wilcox 1982; Riley et al., 2003). The relationship among Fulcidacini genera remains to be studied.

###### Argentinian species checklist.

1. *Exema carinipennis* Monrós, 1951c (COR, MNS).

2. *Exema morio* (Kollar, 1824) (MNS).

= *Chlamys dubia* Kollar, 1824.

= *Chlamys globosa* Klug, 1824 (nec Kollar, 1824). Host plant: Bambuseae (Monrós 1951c).

3. *Exema serjaniae* Monrós, 1951c (CHA, FOR, SAL, TUC). Host plant: Sapindaceae: *Serjania* sp. (Jolivet 1978).

4. *Exema variopicta* Monrós, 1951c (COR, JUY, MNS, SAL, SLS). Host plant: Verbenaceae: *Lantana hypoleuca* and *Lipia salvifolia* (Bokermann 1963).

##### Fulcidax

Taxon classificationAnimaliaColeopteraChrysomelidae

Voet, 1806

Fig. 31

Fulcidax Voet 1806: 33; Jacoby 1880: 90; Monrós 1951c: 641; Blackwelder 1946: 650; Seeno and Wilcox 1982: 43; Chamorro-Lacayo and Konstantinov 2009: 76.
=Poropleura Lacordaire 1848: 863.

###### Type species.

*Fulcidax azureus* Voet, 1806 = *Clytra monstrosa* Fabricius, 1798, by monotypy.

**Figure 31.**
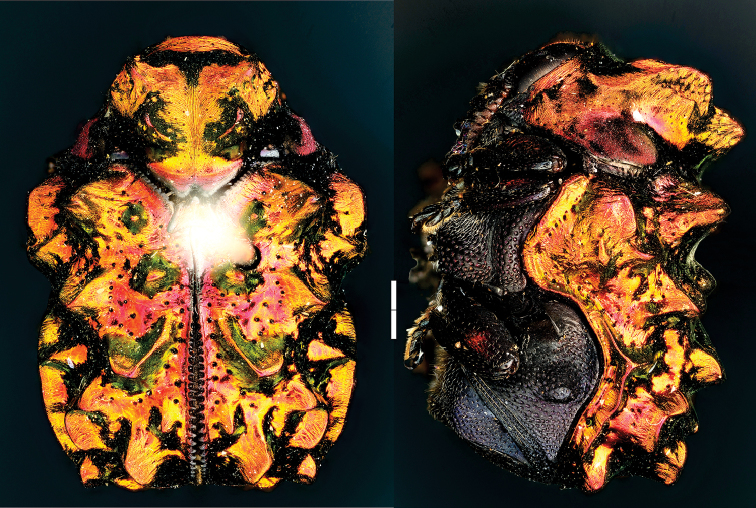
*Fulcidax bacca* (Kirby) ^(2)^, left: habitus (dorsal view), right: habitus (lateral view).

###### Diagnosis.

This genus includes some of the larger and more charismatic species in the group (6.5–7.2 mm length). Body subquadrate and metallic, antenna serrate beyond antennomere III; the head with a longitudinally impressed vertex; elytral tubercles pronounced; posterior pronotal lobe with an acute notch; sutural serration of elytra well-developed beyond the middle of suture towards apex; ventrite I with lateral tubercles; fore- and midtibial apices with spine; tarsal claws simple. According to Chamorro-Lacayo and Konstantinov (2009), *Fulcidax* can be distinguished from all other genera of the tribe by the longitudinally impressed vertex of the head, simple tarsal claws, large body size, and usually bright metallic coloration.

###### Distribution.

From Mexico to Argentina, with seven species.

###### Remarks.

This is a small genus with only seven described species (Monrós 1951c). Chamorro-Lacayo and Konstantinov (2009), mistakenly cited *Fulcidax chimaera* (Lacordaire) for Argentina, this is present in Goiaz state of Brazil.

###### Argentinian species checklist.

1. *Fulcidax bacca* (Kirby, 1818) (CTS, JUY, MNS, SAL, TUC). Host plants: Fabaceae: *Acacia* sp., *Prosopis* sp. (Monrós 1951c); Malpighiaceae: *Mascagnia cordifolia*, *Banisteria stellaris*, *Banisteria argyrophylla* (Bokermann 1963).

##### Melittochlamys

Taxon classificationAnimaliaColeopteraChrysomelidae

Monrós, 1948a

Fig. 32

Melittochlamys Monrós, 1948a: 192; Fiebrig 1910: 253; Monrós 1949b: 617; Monrós 1951c: 666; Seeno and Wilcox 1982: 43; Chamorro-Lacayo and Konstantinov 2009: 80.

###### Type species.

*Chlamys speculum* Klug 1824, by original designation.

**Figure 32.**
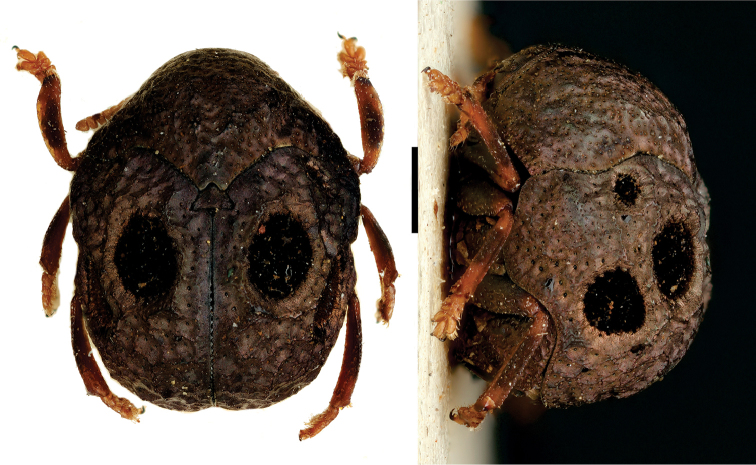
*Melittochlamys specula* (Klug) ^(2)^, left: habitus (dorsal view), right: habitus (lateral view).

###### Diagnosis.

*Melittochlamys* can be separated from all other genera by the nearly rectangular prosternal process; since the process is more or less triangular in all other genera of warty leaf beetles. Intermediate size (length 3.60-5.20 mm); body shape subglobular; antenna serrate beyond antennomere III, antennomere III slightly dilated distally; pronotum without median elevation, relatively smooth; sutural serration of elytra absent or weakly developed; elytra without well developed tubercles; appendiculate tarsal claws.

###### Distribution.

The genus include 13 Neotropical species (Chamorro-Lacayo and Konstantinov 2009).

###### Argentinian species checklist.

1. *Melittochlamys specula* (Klug, 1824) (MNS). Host plant: Myrtaceae: *Psidium guayaba* y *Psidium* sp. (Araça) (Bokermann 1963).

##### Pseudochlamys

Taxon classificationAnimaliaColeopteraChrysomelidae

Lacordaire, 1848

Fig. 33

Pseudochlamys Lacordaire 1848:644; Clavareau 1913: 209; Blackwelder 1946: 647; Monrós 1951c: 542; Karren 1972: 902; Seeno and Wilcox 1982: 43; Chamorro-Lacayo and Konstantinov 2009: 83.

###### Type species.

*Pseudochlamys megalostomoides* Lacordaire 1848, by monotypy.

**Figure 33.**
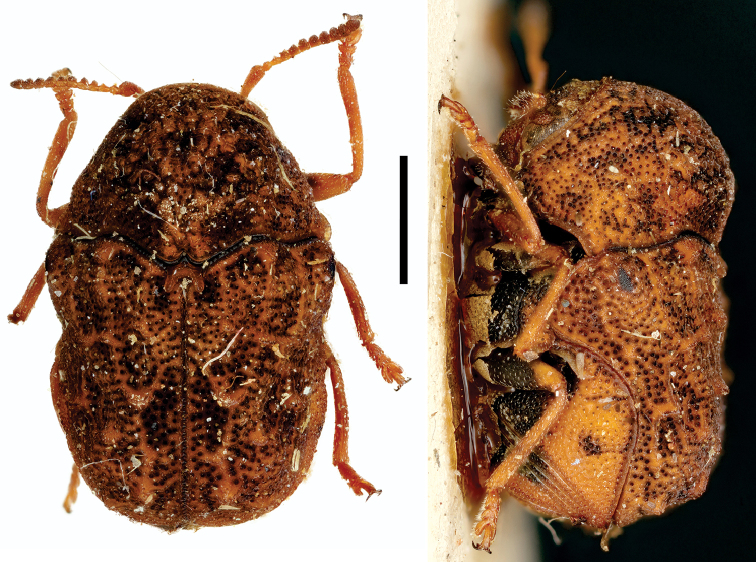
*Pseudochlamys seminigra* (Jacoby) ^(2)^, left: habitus (dorsal view), right: habitus (lateral view).

###### Diagnosis.

*Pseudochlamys* can be distinguished from all other genera in the tribe by: head not completely retracted into prothorax; mandibles enlarged in males (sexual dimorphism); intercoxal prosternal process strongly and abruptly constricted beyond anterior margin; and prosternal process more than ¾ as long as intercoxal prosternal process. These beetles are small sized (length 3.45-4.72 mm), cylindrical; body usually yellowish; canthus of eye as yellow as rest of frons; pronotum and elytra glabrous; head not completely retracted into prothorax; mandibles enlarged in males; antenna serrate beyond antennomere III, antennomere II slightly widened, globose, antennomere V as large as VI; posterior pronotal lobe with well differentiated notch; intercoxal prosternal process strongly and abruptly constricted beyond anterior margin; sutural serration of elytra complete; elytral tubercles poorly developed; tarsal claws bifid or appendiculate.

###### Distribution.

This genus contains only five species, distributed in North, Central, and South America (Chamorro-Lacayo and Konstantinov 2009; Karren 1972).

###### Argentinian species checklist.

*Pseudochlamys seminigra* (Jacoby, 1904) (MNS).

### LAMPROSOMATINAE LACORDAIRE, 1848

Adults: Body compact, strongly convex; head inserted into prothorax (not visible from above). Pronotum convex tightly appressed to elytral base; antennal groove present on each side of prosternal process. Elytra covering pygidium. Larva differs from Cryptocephalinae as pointed out in previous section.

#### Tribe Lamprosomatini Lacordaire, 1848

This tribe is composed of 10 genera (Seeno and Wilcox 1982) and 250 species (Chamorro 2014a). Four genera occur in the Neotropical region (Chamorro 2014a): *Lychnophaes* Lacordaire, *Dorisina* Monrós, *Lamprosoma* Kirby, and *Lamprosomoides* Monrós. It is the only Lamprosomatine tribe represented in Argentina where the fauna comprises 1 genus, *Lamprosoma*, and 8 species. *Lamprosoma* is characterized by the presence of a file on distal margin of last ventrite; last ventrite not excised in shape of arc; pygidium completely covered by elytra; scutellum acutely triangular (small to very small); elytral punctuation arranged in regular rows or with a tendency to form such rows.

##### Lamprosoma

Taxon classificationAnimaliaColeopteraChrysomelidae

Kirby, 1818

Fig. 34

Kirby 1818: 445; Chevrolat in d’Orbigny 1846: 277; Lacordaire 1848: 574; Chapuis 1874: 216; Jacoby 1880: 90; Achard: 1914: 5; Monrós 1948b: 81; Monrós 1956b: 59; Monrós 1960: 9. Caxambú and Almeida 1999: 244; Caxambú and Almeida 2003: 330.

###### Type species.

*Lamprosoma bicolor* Kirby, 1818: 445. By monotypy.

**Figure 34.**
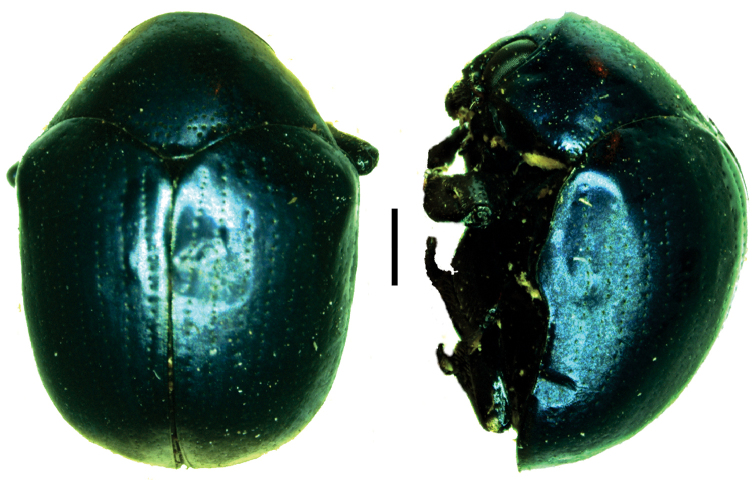
*Lamprosoma azureum* Germar ^(3).^, left: habitus (dorsal view), right: habitus (lateral view).

###### Diagnosis.

body length about 4.5 mm; tarsal claws appendiculate with broad tooth; antenna short, antennomere VIII nearly as wide as VII or IX. Metallic coloration (some species multicolored), head not visible from above, clypeus excavate. According to Monrós (1956b) it can be differentiated from other Neotropical genera by having appendiculate claws at 180º angle, while *Dorisina* and *Lychnophaes* have simple claws at a more obtuse angle.

###### Distribution.

Nearctic, Neotropical, in Argentina limited to north, and northeastern provinces.

###### Remarks.

Adults feed on plants of the families Bombacaceae, Combretaceae, Melastomataceae, Mimosaceae. Therefore, some species have been considered as potential biological control agents for these plants (Caxambú and Almeida 2003).

###### Argentinian species checklist.

1. *Lamprosoma acaciae* Monrós, 1948b (JUY, SAL, TUC). Host plant: Fabaceae: *Acacia* spp. (Bark-gnawing).

2. *Lamprosoma azureum* Germar 1824 (MNS). This species newly cited for Argentina.

3. *Lamprosoma chorisiae* Monrós, 1948b (CHA).

= *Lamprosoma chaguanacum* Monrós, 1948b. Parasitoids: Gelini and Hemitelini (Ichneumonidae) (Monrós 1948b). Host plant: Bombacaceae: *Chorisia* sp. (Monrós 1948b).

4. *Lamprosoma indigaceum* Monrós, 1947 (CTS).

5. *Lamprosoma minimum* Monrós, 1948b (SAL). Host plant: Fabaceae: *Acacia cavenia* (Bark-gnawing).

6. *Lamprosoma subnitidum* Monrós, 1948b (CTS).

7. *Lamprosoma triste* Guérin-Ménéville, 1844 (Northeast Argentina).

8. *Lamprosoma zariateguii* Monrós, 1947 (MNS).

## Discussion and conclusions

This is the first comprehensive synthesis of Argentinian Camptosomata. This study may prove useful also for countries bordering Argentina. Similar contributions indicated the diversity of Camptosomata in other Neotropical countries as follows: Maes (1998) recorded 19 genera and 46 species for Nicaragua, Chaboo and Schöller (2016) accounted for 14 genera and 43 species for Peru; and in Brazil, 723 species, 26 subspecies in 37 genera of Cryptocephalinae (Sekerka et al. 2015) and 62 species in 5 genera of Lamprosomatinae (Sekerka 2017) were recorded.

### Species richness and distribution patterns

**Figure 35.**
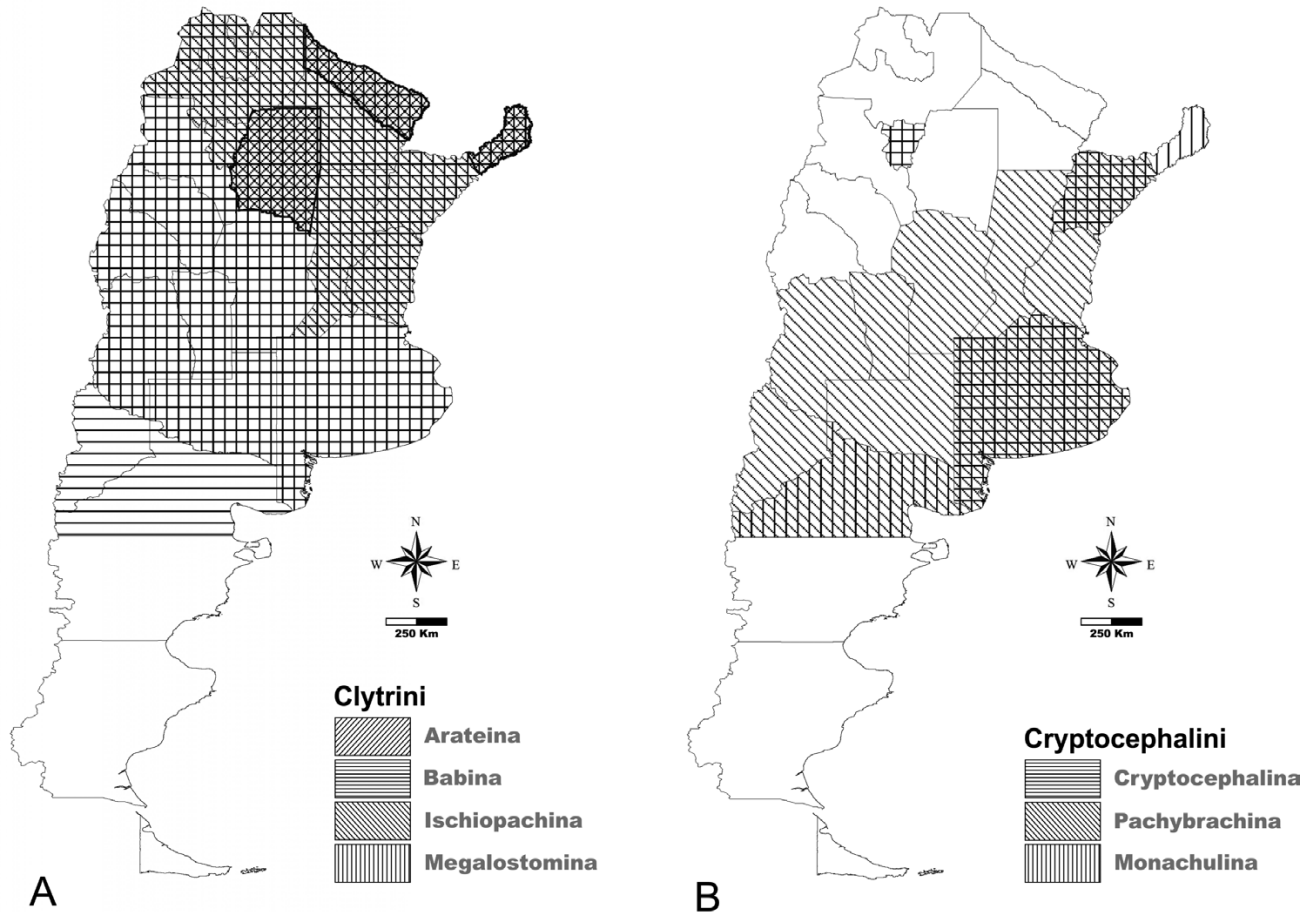
**A** Administrative divisions representing presence of subtribes of Clytrini as indicated in reference **B** Administrative divisions indicating presence of subtribes of Cryptocephalini as indicated in reference.

**Figure 36.**
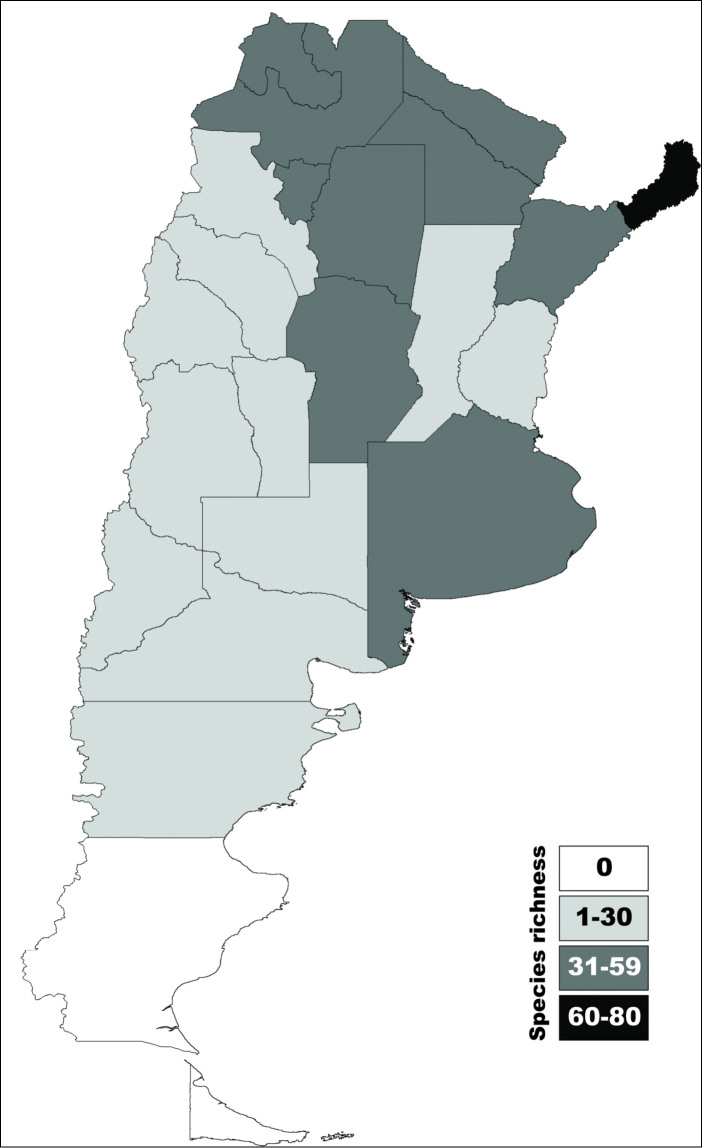
Map indicating species number (0–80) (richness) by province.

**Figure 37.**
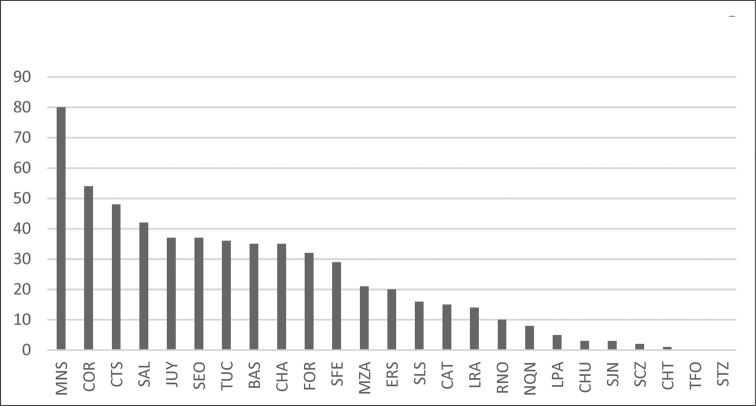
Bars diagram showing species by provinces, it can be observed that species richness diminished through southern provinces.

Historically, Argentina has been divided in two main regions: Andean and Neotropical (Morrone, 2014). As depicted in the distribution pattern of Camptosomata tribes and subtribes by province (Fig. 35A–D), tribes are mostly distributed in the Neotropical region, while few species reach the Andean region or are found below 40º S latitude. Based on the map of species richness by province (Fig. 36) higher richness (up to 80 species) roughly coincides with the line dividing the Neotropical and Andean regions (Figs 1, 37). Poor data notwithstanding, this pattern fits at the latidudinal diversity gradients hypothesis with greater species richness at tropical latitudes (Hillebrand 2004). Most of the central and northern provinces (*e.g.* CHA, COR, CTS, FOR, MNS, SAL, SEO, TUC) are presented on the left side of Figure 37, while most of southern provinces (CHT, CHU, NQN, SCZ, STZ, and TFO) appear on the right side, with few exception, for example the lack of information for SJN, ERS, or SLS.

Within Clytrini (Fig. 35A), Arateina is present in the northeastern provinces (FOR, MNS, SEO), while Ischiopachina is distributed throughout most of northeastern Argentina. Megalostomina is present from northern Argentina to the central region (as far as MZA, LPA, and BAS). Babiina covers this same region, yet it reaches the southern provinces (NQN, and RNO). Clytrini has not been reported for the southern provinces beyond Rio Negro.

Cryptocephalini, on the other hand, is putatively mostly absent from the Northwestern provinces of Argentina. The presence of this tribe in Tucumán might indicate a more widespread distribution. Sampling bias and poor inventory may explain the absence of Fulcidacini in central and western regions of Argentina. The subtribes of Cryptocephalini (Fig. 35B) show a more widespread distribution for Cryptocephalina and Pachybrachina, while Monachulina are mostly recorded from Northeastern Argentina. Finally, Lamprosomatinae seems restricted to the Northern provinces, its absence in Formosa seems artificial, so presence of this subfamily surely will expand with more collecting in this region. According to current information, most species are distributed in the Neotropical provinces, especially: Araucaria forest, Chacoan, Monte, Pampean, Parana Forest, and Prepuna.

### Current taxonomic knowledge, basic statistics and future research

**Figure 38.**
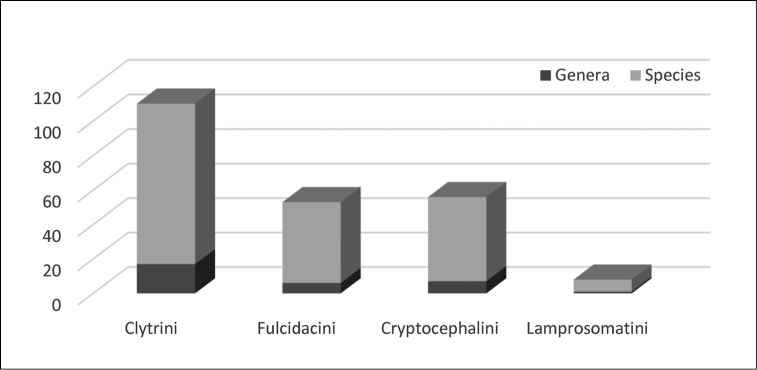
Bars diagram showing the number of genera and species by tribe of Argentinian Camptosomata.

**Figure 39.**
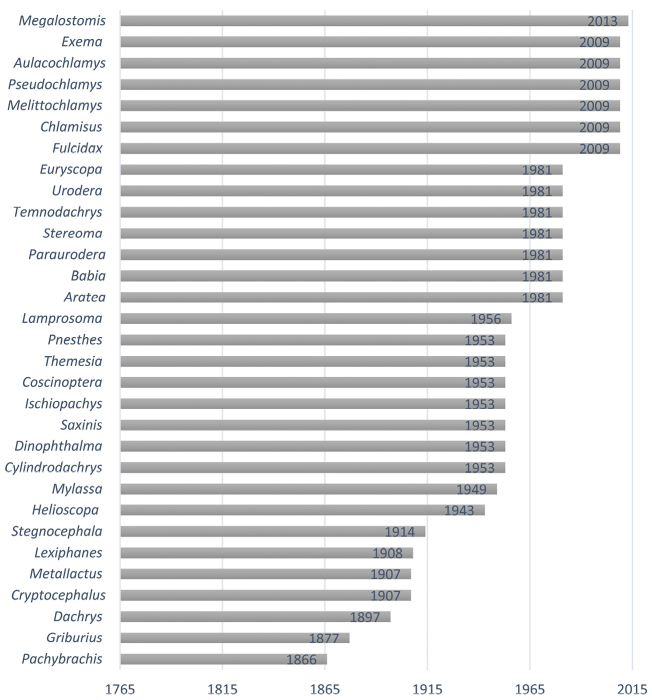
Timelines showing the years of the last work made on each genera that includes Argentinian species taxonomic treatment (simple checklist included).

**Figure 40.**
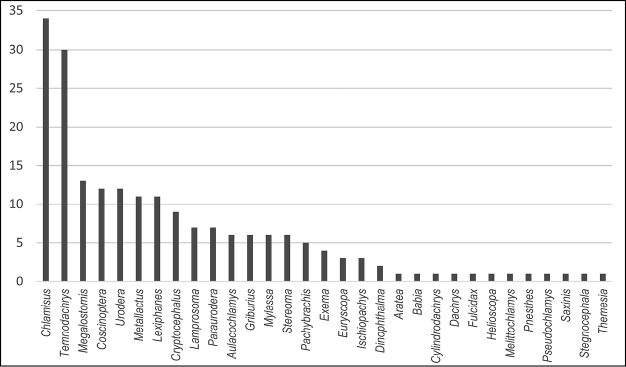
Species number by genus.

A total of 190 species (182 Cryptocephalinae + 8 Lamprosomatinae) of Camptosomata are currently known from Argentina. The most diverse group of Camptosomata in Argentina is Clytrini (Fig. 38). However, Clytrini is also, by far, the most studied group in Argentina due to the efforts of Monrós in the 1950’s. The patchy distribution at administrative division levels clearly indicates the need for specimen identification and incorporation of museum specimens into databases, as well as collection of new specimens. The latter will permit the application of ecological modelling and biogeographic studies of the group that will provide a more complete picture of the biogeographic history and ecological tolerance ranges, as well as help guide conservation policies for the group. The current estimate of endemic species in Argentina is uncertain, and its calculation based on extant information would be inaccurate, especially without a complete species checklist of bordering countries (i.e. Bolivia, Brazil, Chile, Paraguay, and Uruguay). When comparing the timelines in Fig. 39 with the graphic indicating species richness by genus (Fig. 40), it becomes clear that (except for Megalostomis, recently revised by Agrain (2013), several of the most diverse genera have not been revised in over 100 years. Many species are only known from their original descriptions in the mid 19th or mid 20^th^ century (Fig. 39). In many cases, the type specimens were not illustrated. This has resulted in long series of unidentified specimens housed in public and private collections awaiting the study of name bearing types.

Our synthesis here is a necessary step towards further comprehensive study of Argentinian Camptosomata that will facilitate field work, assist in determination of unidentified material housed in South American collections, creation of illustrated of keys to the species level, and with identified specimens in hand achieve databasing of museum specimens. These elementary tasks are prerequisite to modern taxonomic revisions and evolutionary studies.

## Supplementary Material

XML Treatment for Aratea

XML Treatment for Babia

XML Treatment for Babia (Coleolacordairei)

XML Treatment for Cylindrodachrys

XML Treatment for Dachrys

XML Treatment for Dinophthalma

XML Treatment for Helioscopa

XML Treatment for Paraurodera

XML Treatment for Paraurodera (Paraurodera)

XML Treatment for Paraurodera (Torourodera)

XML Treatment for Pnesthes

XML Treatment for Stereoma

XML Treatment for Saxinis

XML Treatment for Saxinis (Saxinis)

XML Treatment for Temnodachrys

XML Treatment for Temnodachrys (Eudachrys)

XML Treatment for Temnodachrys (Temnodachrys)

XML Treatment for Urodera

XML Treatment for Urodera (Austrurodera)

XML Treatment for Urodera (Stereomoides)

XML Treatment for Ischiopachys

XML Treatment for Coscinoptera

XML Treatment for Euryscopa

XML Treatment for Euryscopa (Coleomonrosa)

XML Treatment for Megalostomis

XML Treatment for Themesia

XML Treatment for Cryptocephalus

XML Treatment for Lexiphanes

XML Treatment for Stegnocephala

XML Treatment for Griburius

XML Treatment for Metallactus

XML Treatment for Mylassa

XML Treatment for Pachybrachis

XML Treatment for Aulacochlamys

XML Treatment for Chlamisus

XML Treatment for Exema

XML Treatment for Fulcidax

XML Treatment for Melittochlamys

XML Treatment for Pseudochlamys

XML Treatment for Lamprosoma
